# Nanoscale Phosphoinositide Distribution on Cell Membranes of Mouse Cerebellar Neurons

**DOI:** 10.1523/JNEUROSCI.1514-22.2023

**Published:** 2023-06-07

**Authors:** Kohgaku Eguchi, Elodie Le Monnier, Ryuichi Shigemoto

**Affiliations:** Institute of Science and Technology Austria (ISTA), Am Campus 1, Klosterneuburg 3400, Austria

## Abstract

Phosphatidylinositol-4,5-bisphosphate (PI(4,5)P_2_) plays an essential role in neuronal activities through interaction with various proteins involved in signaling at membranes. However, the distribution pattern of PI(4,5)P_2_ and the association with these proteins on the neuronal cell membranes remain elusive. In this study, we established a method for visualizing PI(4,5)P_2_ by SDS-digested freeze-fracture replica labeling (SDS-FRL) to investigate the quantitative nanoscale distribution of PI(4,5)P_2_ in cryo-fixed brain. We demonstrate that PI(4,5)P_2_ forms tiny clusters with a mean size of ∼1000 nm^2^ rather than randomly distributed in cerebellar neuronal membranes in male C57BL/6J mice. These clusters show preferential accumulation in specific membrane compartments of different cell types, in particular, in Purkinje cell (PC) spines and granule cell (GC) presynaptic active zones. Furthermore, we revealed extensive association of PI(4,5)P_2_ with Ca_V_2.1 and GIRK3 across different membrane compartments, whereas its association with mGluR1α was compartment specific. These results suggest that our SDS-FRL method provides valuable insights into the physiological functions of PI(4,5)P_2_ in neurons.

**SIGNIFICANCE STATEMENT** In this study, we established an electron microscopic method to visualize and analyze the quantitative distribution pattern of phosphatidylinositol-4,5-bisphosphate (PI(4,5)P_2_) on cell membranes using cryo-fixed brain tissues and SDS-digested freeze-fracture replica labeling. PI(4,5)P_2_ interacts with various ion channels and receptors to regulate membrane signaling but its nanoscale distribution and association with these proteins remain elusive. This method revealed PI(4,5)P_2_ clusters preferentially accumulated in specific membrane compartments and its distinct associations with Ca_V_2.1, GIRK3, and mGluR1α in the mouse cerebellum. These results demonstrate usefulness of the method for gaining insights into the physiological functions of PI(4,5)P_2_.

## Introduction

Phosphoinositides (PIs) are minor components on the cytoplasmic side of eukaryotic cell membranes, but they play essential roles in a wide variety of cellular functions. In neuronal cells, each stereoisomer of PIs is distributed in different subcellular compartments (Wenk and De Camilli, 2004; Haucke, 2005; Idevall-Hagren and De Camilli, 2015): PI(4)P is enriched in the membrane of the Golgi apparatus and synaptic vesicles (SVs), PI(4,5)P_2_ and PI(3,4,5)P_3_ mainly exist in the plasma membrane, PI(3)P and PI(3,5)P_2_ are selectively concentrated on early and late endosomes, respectively. PIs contribute to various aspects of neuronal activity, such as synaptic transmission and maintenance of membrane excitability by regulating ion channels and intracellular signaling pathways. At chemical synapses, PIs regulate exocytosis and endocytosis of synaptic vesicles at the presynaptic sites (T.F.J. Martin, 2001; Ueda and Hayashi, 2013; Posor et al., 2015; Lei et al., 2017). Many presynaptic proteins involved in such regulation have binding domains to stereoisomers of PIs (Di Paolo and De Camilli, 2006; Falkenburger et al., 2010), indicating their potential role as an anchor for these proteins to regulate their localization and dynamics during synaptic transmission. At the postsynaptic sites, PIs regulate plasticity of dendritic spines through actin remodeling during long-term potentiation (T.F.J. Martin, 2001; Ueda and Hayashi, 2013; Posor et al., 2015; Lei et al., 2017).

The distribution pattern of PIs and their association with signaling proteins at neuronal cell membranes are crucial for understanding their roles in neuronal activities, but have been poorly investigated because of several technical issues with the conventional methods (Tsuji et al., 2019). For example, immunostaining with an anti-PI(4,5)P_2_ antibody in aldehyde-fixed cell preparations may not show actual phospholipid distribution because aldehydes cannot fix the lateral diffusion of most membrane lipids (K.A.K. Tanaka et al., 2010). For live cell imaging of PIs, fluorescent protein tagged PI-binding domain (PBD) has been used as specific PI probes (Maekawa and Fairn, 2014; Idevall-Hagren and De Camilli, 2015). However, this method has insufficient spatial resolution to observe the nanoscale PIs distribution in small membrane compartments, such as presynaptic active zones (AZs) and postsynaptic densities (PSDs). In addition, the overexpressed PBD-based probes mask PIs and competitively interfere with their interactions with proteins (Suh and Hille, 2008).

To solve these issues and visualize the nanoscale distribution of PIs on cell membranes, sodium dodecyl sulfate-digested freeze-fracture replica labeling (SDS-FRL) combined with cryofixation has been used (Fujita et al., 2009; Ozato-Sakurai et al., 2011; Cheng et al., 2014; Aktar et al., 2017; Tsuji et al., 2019). This electron microscopic method enables nanoscale phospholipid visualization with physically fixed PIs by high-pressure freezing and carbon/platinum replication. In this study, we optimized this SDS-FRL method and investigated the nanoscale distribution of PI(4,5)P_2_ on mouse cerebellar neuronal membranes using recombinant GST-tagged pleckstrin homology (PH) domain of phospholipase Cδ1 (PLCδ1) as a specific PI(4,5)P_2_ probe. This approach allowed us to examine the numbers, densities, and distribution patterns of PI(4,5)P_2_ on somatodendritic and axonal membranes, including postsynaptic and presynaptic sites. We show that PI(4,5)P_2_ makes small clusters on neuronal membranes and specifically co-clusters with P/Q-type voltage-gated calcium channels, G-protein-coupled inwardly rectifying potassium channels, and metabotropic glutamate receptors in distinct membrane compartments, giving insights into the physiological functions of PI(4,5)P_2_ in the regulation of neuronal excitability and neurotransmitter release.

## Materials and Methods

### Animals

Animal experiments were conducted in accordance with the guideline of the Institute of Science and Technology Austria (Animal license number: BMWFW-66.018/0012-WF/V/3b/2016). Male C57BL/6J (stock #000664) mice at postnatal (P) five to seven weeks were used in this study. Mice were initially purchased from The Jackson Laboratory and were bred at the Preclinical Facility of IST Austria on 12/12 h light/dark cycle with access to food and water *ad libitum*. All experiments were performed in the light phase of the cycle.

### Antibodies

Table 1 shows a list of the primary antibodies containing their epitopes, concentrations for use, suppliers, and specification that were used in this study. Gold particle-conjugated secondary antibodies were purchased from British Biocell International (BBI, goat anti-rabbit IgG, 5 nm; goat anti-guinea pig IgG, 10 nm; goat anti-mouse IgG, 15 nm) and Jackson ImmunoResearch (donkey anti-chicken IgY, 6 nm; donkey anti-guinea pig IgG, 12 nm).

**Table 1.** Antibody list for SDS-FRL

Molecule	Catalog #	Host	Supplier	Epitope	RRID	Lot	Conc.	Specificity	Reference
GST	A190-122A	Rb	Bethyl	—	AB_67419	#11	5 µg/ml	W/o antigen	Present study
GST	A190-123A	Ck	Bethyl	—	AB_66670	#2	5 µg/ml	W/o antigen	Present study
RFP	M155-3	Ms	MBL	—	AB_1278880	#016	2 µg/ml	W/o antigen	Present study
Ca_V_2.1	152 205	Gp	SYSY	Rt 1921-2212aa	AB_2619842	#1–5	2.5 µg/ml	KO, FRL	Eguchi et al. (2022)
GluD2	MSFR102610	Gp	FI	Ms 897-934aa	AB_2571603	—	2 µg/ml	KO, IF	Konno et al. (2014)
GIRK3	MSFR102100	Rb	FI	Ms 358-389aa	AB_2571714	—	4 µg/ml	KO, FRL	Luján et al. (2018b)
mGluR1α	MSFR104080	Gp	FI	Ms 945-1127aa	AB_2571801	—	2 µg/ml	KO, FRL	Mansouri et al. (2015)

Rb, rabbit; Ck, chicken; Ms, mouse; Gp, guinea pig; Rt, rat; SYSY, Synaptic Systems; FI, Frontier Institute; KO, knock-out; FRL, SDS-digested freeze-fracture replica labeling; IF, immunofluorescence; -, information is not provided by suppliers.

### Liposome preparation and high-pressure freezing

Phosphatidylcholine (18:1 (Δ9-Cis) PC), phosphatidylethanolamine (18:1 (Δ9-Cis) PE), phosphatidylserine (18:1 (Δ9-Cis) PS), phosphatidylinositol (18:1 PI), and phosphoinositides (18:1) were purchased from Avanti Polar Lipids. All liposomes contained 45 mol % PC, 30 mol % PE, 20 mol % PS, and either 5 mol % PI or a phosphoinositide. When preparing liposomes containing various concentration of PI(4,5)P_2_, the total concentration of PC and PI(4,5)P_2_ was adjusted to 50 mol%. Solutions of PC, PE, and PS in chloroform and PI or phosphoinositides in chloroform:methanol:H_2_O:HCl (1 N; 20:9:1:0.1) were mixed in the required proportion in amber-color glass vials. To improve phosphoinositide homogenization with other lipids, the chloroform:methanol 2:1 ratio was maintained in the mixture. A lipid film was produced by evaporation of solvents in the vial under the stream of nitrogen gas and then drying using a vacuum desiccator for 2 h. The dried lipid film was stored at −20°C with argon gas and used within 2 d.

The lipid film was resuspended in a buffer containing 220 mm sucrose and 20 mm HEPES (pH 7.4, adjusted with NaOH). The suspension was vortexed well and then freeze-thawed 5 times in liquid nitrogen and warm water (∼60°C). Unilamellar liposomes were produced by extrusion through a 0.4-µm pore size polycarbonate filter using an extrusion apparatus (Avanti Polar Lipids). After extrusion, the liposomes were diluted five times in 120 mm NaCl, 20 mm HEPES buffer (pH 7.4, adjusted with NaOH), and centrifuged for 10 min at 10,000 × *g*. The supernatant was carefully removed, and glycerol was applied as a cryoprotectant to the liposome pellet to a final concentration of 50%. The liposome/glycerol mixture was placed on a copper carrier with a ring of double-sided tape (140-µm thickness), covered with another carrier, and then frozen by a high-pressure freezing machine (HPM010, BAL-TEC). The frozen samples were stored in liquid nitrogen until use.

### Cell culture, transfection, and high-pressure freezing of HEK293 cells

The cDNA pCAGG PM-FRB-mRFP-T2A-FKBR-5-ptase was constructed by adding the PM-FRB-mRFP-T2A-FKBP-5-ptase fragment (a gift from Peter Varnai, Addgene, #40896; http://n2t.net/addgene:40896; RRID:Addgene_40896; Tóth et al., 2012) to pCAGG vector from our own library. Plasmid DNA was purified on a Nucleobond AX anionexchange column (Macherey-Nagal).

Human Embryonic kidney 293 (HEK293) cells were seeded at a density of 2 × 10^6^ cells per 100-mm dish and maintained in DMEM supplemented with 10% fetal bovine serum, 100 U/ml penicillin and streptomycin (Invitrogen) in a humidified atmosphere (5% CO_2_) at 37°C. HEK293 cells were transiently transfected using Lipofectamine 3000 (ThermoFisher Scientific) according to the manufacturer's protocol. Forty-eight hours after transfection, cells were treated with 0.25% trypsin-EDTA (Sigma-Aldrich) for 2–5 min at 37°C and collected by centrifugation (100 × *g*, 5 min). After removing supernatant, cells were incubated with rapamycin (5 μm with 0.1% DMSO) or 0.1% DMSO dissolved in PBS for 5 min at room temperature (RT) and centrifuged for 5 min at 100 × *g*. The supernatant was carefully removed, and the pellet was placed on a copper carrier with a ring of double-sided tape (140-µm thickness), covered with another carrier, and then frozen by a high-pressure freezing machine. The frozen samples were stored in liquid nitrogen until use.

### High-pressure freezing of acute cerebellar slices

Acute slices of mouse cerebellum were prepared at physiological temperature (PT) to avoid the alternation of the neuronal conditions, such as profound loss of dendritic spines and synaptic proteins, as described previously (Eguchi et al., 2020). Briefly, mice were decapitated under isoflurane anesthesia and their brains were quickly removed from the skull and immersed into a cutting solution containing (mm): 300 sucrose, 2.5 KCl, 10 glucose, 1.25 NaH_2_PO_4_, 2 Na Pyruvate, three *myo*-inositol, 0.5 Na ascorbate, 26 NaHCO_3_, 0.1 CaCl_2_, 6 MgCl_2_ (pH 7.4 when gassed with 95% O_2_/5% CO_2_) at PT (35–37°C). The cerebellum was dissected from the whole brain and immediately glued on a cutting stage of a tissue slicer (Linear Slicer Pro7, Dosaka EM) and sliced (sagittal, 140–160 µm thickness) in the cutting solution kept at PT. Slices were then maintained in the artificial cerebrospinal fluid (ACSF) containing (in mm): 125 NaCl, 2.5 KCl, 10 glucose, 1.25 NaH_2_PO_4_, 2 sodium pyruvate, three *myo*-inositol, 0.5 sodium ascorbate, 26 NaHCO_3_, 2 CaCl_2_, 1 MgCl_2_ (pH 7.4 when gassed with 95% O_2_/5% CO_2_) at 37°C until use. Small blocks containing lobule IV–VII were trimmed from the slices in the cutting solution using a micro scalpel (#10316-14, FST) and transferred into cryoprotectant buffer [15% polyvinylpyrrolidone (PVP) in ACSF with 10 mm HEPES, pH 7.3 adjusted with NaOH], sandwiched between two copper carriers with a ring of double-sided tape (140-µm thickness), and then frozen by a high-pressure freezing machine. The frozen samples were stored in liquid nitrogen until use. We froze the acute cerebellar slices within 2 h after slicing to ensure that the slice condition did not alter (Eguchi et al., 2020).

### SDS-digested freeze-fracture replica labeling (SDS-FRL)

The frozen samples were fractured into two parts at −130°C and replicated by carbon (4–5 nm thick), carbon-platinum (uni-direction from 60°, 2 nm), and carbon (20–25 nm) deposition in a freeze-fracture machine (JFD-V, JOEL). The samples were digested with 2.5% SDS in 0.1 m Tris-HCl (pH 8.3) at 80°C for 18–22 h. The replicas were washed in the SDS solution and then a washing buffer (50 mm Tris-buffered saline (TBS; pH 7.4) containing 0.1% BSA) at RT. To avoid nonspecific binding of the probes and antibodies, the replicas were blocked with 3% BSA, 2% cold fish skin gelatin, and 0.05% Tween 20 in TBS for 1 h at RT. The replicas were incubated with 50 ng/ml GST-tagged PH domain of phospholipase C δ1 (PI(4,5)P_2_-Grip, Echelon Inc.) in a dilution buffer (1% BSA, 1% cold fish skin gelatin, and 0.05% Tween 20 in TBS) at 4°C overnight. Then the replica was incubated with anti-GST antibody and anti-Ca_V_2.1 antibody as a marker of neurons at 15°C overnight, and then gold-nanoparticle conjugated secondary antibodies dissolved in the dilution buffer at 15°C overnight. For double labeling of PI(4,5)P_2_ with proteins, the replicas were incubated with a mixture of primary antibodies (GluD2: rabbit anti-GST + guinea pig anti-GluD2; GIRK3: chicken anti-GST + rabbit anti-GIRK3; mGluR1α: rabbit anti-GST + guinea pig anti-mGluR1α) at 15°C 1–2 overnight and then with gold-nanoparticle conjugated secondary antibodies at 15°C overnight. After washing the replicas with the washing buffer, they were picked up onto a grid coated with formvar in distilled water. Images were obtained under TEM (Tecnai 10) operated at 80 kV with RADIUS software at magnifications of 65,000 and 39,000.

### Image analysis

Images were analyzed with Darea software (Kleindienst et al., 2020), Fiji (Schindelin et al., 2012), and R. The gold particle detection and the demarcation of the region of interest were performed on Darea software. AZs on the P-face of PF boutons were indicated with the aggregation of intramembrane particles on the replica at the electron microscopic level as described previously (Landis and Reese, 1974; Harris and Landis, 1986; Masugi-Tokita et al., 2007; Eguchi et al., 2020, 2022). Gold particles inside or <30 nm away from the demarcation border of AZs (outer rim) were counted as the particles in the AZs (Kleindienst et al., 2020). Because the PSD area on the P-face of dendritic spines of PCs cannot be identified based on morphologic features, the largest cluster of GluD2-labeling gold particles on the spines was identified as the PSD area (Konno et al., 2014; Luján et al., 2018a; Eguchi et al., 2020). The demarcated region of the images was imported to R via FIJI/ImageJ for the following point pattern analysis.

Point pattern analysis of the gold particles described below was performed using spatstat package (version 2.3–0) of R (version 4.1.0). Nearest neighbor distances (NNDs) to both particles of the same size (e.g., from a 5-nm particle to the nearest 5-nm particle) and the other size (e.g., from a 5-nm particle to the nearest 10-nm particle) were computed to evaluate the distribution pattern of the particles. Center Periphery Index (CPI), indicating the location of the particles in the AZs or PSDs, was calculated as the square of the normalized distances from the center of the region of interest. When particles are randomly distributed in a circle, the mean CPI is near 0.5 (Kleindienst et al., 2020). To assess the randomness of the particle distribution, we performed two types of Monte-Carlo simulations, termed random and fitted simulations, following the methods described in the previous publications (Luján et al., 2018b; Kleindienst et al., 2020) using R. For the random simulation, particles were randomly placed on the demarcated area. The simulated particles were placed to keep the minimum distance of 10 nm from any other particles and then randomly shifted within a disk with a 30-nm diameter to reproduce the immunolabeling with a probe and antibodies (Tabata et al., 2019). In the fitted simulation, a constraint was added that the distance distribution between the simulated particles should not differ significantly from the distance distribution between the original particles. The particle distribution pattern was modeled and simulated as a Matern Cluster point process, and the goodness-of-fit between the real and simulated distribution was assessed by comparing both the all pairwise distances (APD) and NNDs of the particles using the two-sample Kolmogorov–Smirnov test (KS test), respectively, and considered them similar if the *p*-value was equal or above 0.1 for both. To avoid excessive statistical power because of the large sample size caused by a large number of particles, parametric bootstrapping was performed when the number of values to be compared exceeded 100. Specifically, we first randomly selected 100 distance values (NND or APD) from the simulated distribution and compared them to the empirical cumulative distribution function of the distance values of the real distribution using the KS test. This process was repeated 1000 times, and the average of the *p*-values was used to assess the goodness-of-fit.

Gold particle clusters were detected with a hierarchical clustering algorithm called Ward linkage and a density-based clustering algorithm called DBSCAN. For Ward linkage, the threshold distance was set as 50 nm. For DBSCAN, we set the minimum number of particles consisting of a cluster as three and the maximum distance between particles as the sum of the median and 1.5 times the interquartile range (IQR) of the NNDs. The area of the convex polygon connecting the outermost particles forming the cluster was defined as the cluster area.

### Estimation of the labeling efficiency

**Figure 1.**
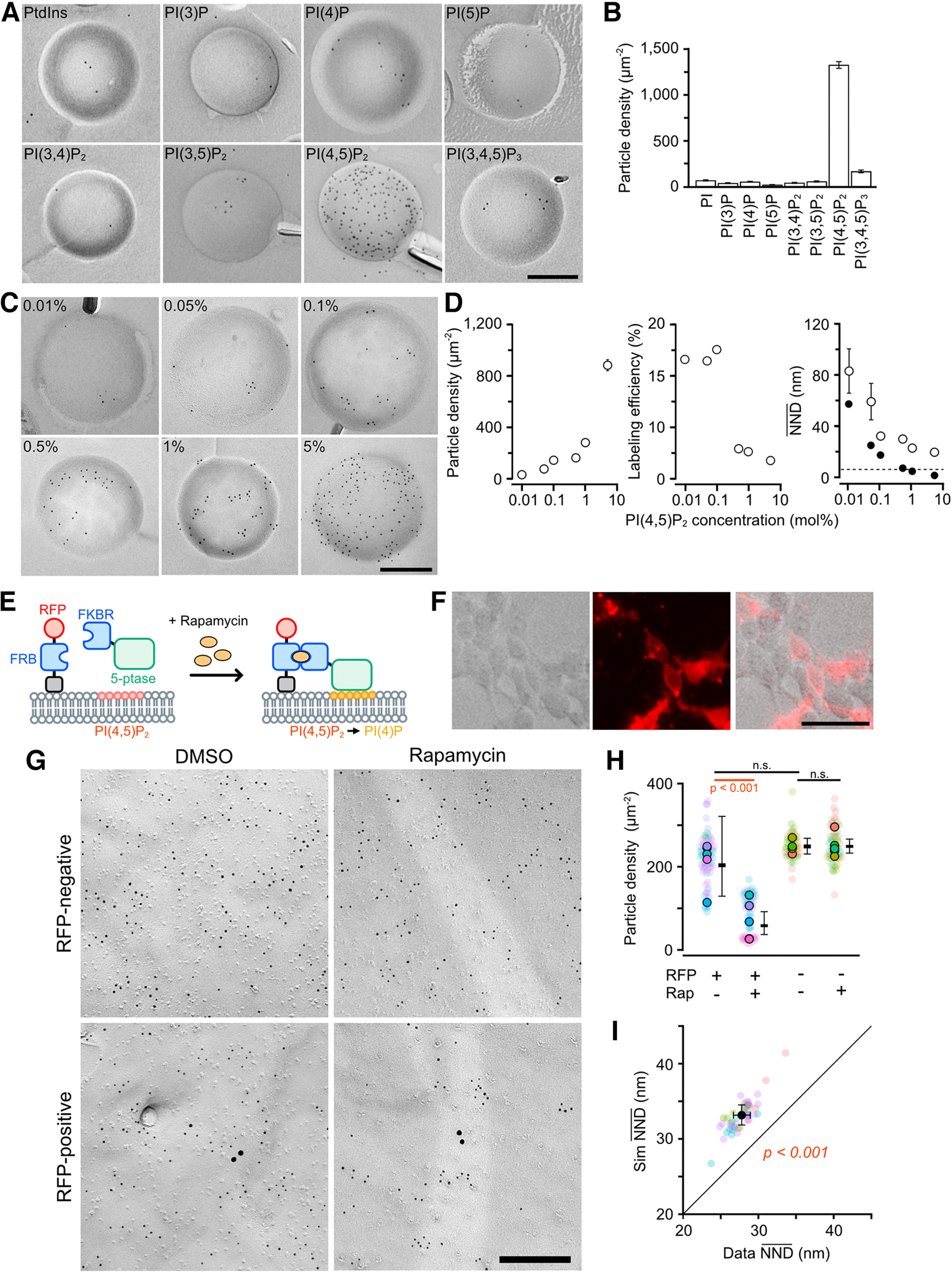
PI(4,5)P_2_ labeling using SDS-FRL. ***A***, Example transmission electron microscopic (TEM) images of liposome replicas containing 5% PtdIns or different stereoisomers of PIs labeled with GST-PH, anti-GST antibody, and 5-nm gold particle-conjugated secondary antibody. Scale bar = 200 nm. ***B***, Bar graph indicating the gold particle density on the liposome replicas. The density of gold particles was the highest in the liposome containing PI(4,5)P_2_. ***C***, Example TEM images of liposome replicas containing different concentration of PI(4,5)P_2_. PI(4,5)P_2_ was labeled with 5-nm gold particles. Scale bar = 200 nm. ***D***, Concentration-dependent changes of the PI(4,5)P_2_ labeling. Left, The gold particle densities in the liposome replica. Middle, The labeling efficiency for PI(4,5)P_2_. Right, The mean NNDs of the observed gold particles (open) and theoretical mean NNDs of randomly distributed PI(4,5)P_2_ molecules (black). Dashed line indicates the diameter of PLCδ1-PH. Error bars indicate SEM. ***E***, Schema of chemically-inducible PI(4,5)P_2_ depletion with FRB-FKBR system. Rapamycin induces heterodimerization of FRB and FKBR, causing translocation of 5-ptase on the cell membrane. ***F***, Example image of HEK293 cells transfected with PM-FRB-mRFP-TA-FKBR-5-ptase (left, bright field; middle, mRFP fluorescent; right, merged). Scale bar = 50 µm. ***G***, Example TEM images of 5-nm gold particle labeling for PI(4,5)P_2_ on the P-face of the somatic membranes of HEK293 cells treated with rapamycin (5 µM) or vehicle (0.1% DMSO). The somatic membranes of the transfected cells were identified by immunogold labeling for RFP (15 nm). Scale bar = 200 nm. ***H***, Statistical comparison of the PI(4,5)P_2_ particle density on the somatic membranes of HEK293 cells. Closed and transparent circles indicate the means of PI(4,5)P_2_ particle density in each cell and each image, respectively, with colors indicating different cells. Horizontal bars and error bars indicate estimated marginal means (emmeans) and 95% confidence intervals (CIs) of the density estimated by GLMM (see Materials and Methods). The density on the rapamycin-treated transfected cells was significantly lower than others. ***I***, Comparison of nearest neighbor distances (NND) between real (Data NND, *x*-axis) and simulated (Sim NND, *y*-axis) PI(4,5)P_2_ particles on the somatic membranes of the untransfected HEK293 cells treated with DMSO. Data-NNDs are significantly smaller than Sim NNDs [Data: 27.8 ± 0.6 nm, Sim: 33.2 ± 0.7 nm, *n* = 49 images/4 cells, *p* < 0.001, likelihood ratio χ^2^ test (Chi-LRT)]. n.s., not significant.

The labeling efficiency for PI(4,5)P_2_ with GST-PH was estimated on liposome replicas containing 0.01–5 mol% PI(4,5)P_2_. Considering that a single phospholipid molecule occupies a space of ∼0.65 nm^2^ (Nagle and Tristram-Nagle, 2000), 750,000 phospholipids would construct a half leaflet of a 1 µm^2^ lipid bilayer. Background gold particle density (20 particles/µm^2^; Fig. 1*B*) was subtracted from the gold particle density on the liposomes. The labeling efficiency for each PI(4,5)P_2_ concentration was estimated by dividing the gold particle density by the theoretical PI(4,5)P_2_ density. For example, a 5 mol% PI(4,5)P_2_-containing liposome replica has a theoretical density of 37 500 molecules/µm^2^, thus the labeling efficiency is estimated to be 2.3% based on the gold particle density of 850 particles/µm^2^. Assuming random distribution of PI(4,5)P_2_ on the liposomes, the expected mean NND ($\bar{NND}$) of the molecules is given as:

(1) $$\bar{NND}=\frac{0.5}{\sqrt{D}},$$

where *D* is the density of the molecules in the area.

### Statistical analysis

To consider the hierarchical structure, correlation, and probability distribution, data were analyzed with either a linear mixed-effects model (LMM) or its generalized form (GLMM) using the lme4 package (version 1.1–27.1) of R (Aarts et al., 2014; Yu et al., 2022). Probability distributions for models were chosen by the goodness of fit to Poisson (for discrete variables e.g., the number of particles), normal (e.g., CPI), or the gamma distribution (for continuous variables e.g., NNDs). Appropriate to the particular experiment and statistical model, treatments (transfection, rapamycin-application), the faces of cell membranes (P- and E-face), components of neurons (e.g., somata, spines, AZs on PF boutons), and potentially their interactions were used as fixed effects, while experiments, animals, replicas, and cells were used as random effects to consider the nested and/or crossed data structure in the statistical analysis. For all experiments, at least four animals per condition were used. All data are presented as estimated marginal means (emmeans) with 95% confidence intervals (CIs, in figures) or SEM (in text) estimated using the emmeans package. The goodness-of-fit of the models was assessed by second-order Akaike Information Criterion (AICc) for the elimination of the random factors (MuMIn package) and then by likelihood ratio χ^2^ tests (Chi-LRT) with models in which the fixed effects of interest had been dropped. For multiple pairwise comparisons between three or more groups, *post hoc* comparisons to assume the significance of differences between pairs of group means were performed using emmeans packages with Tukey (for all pairwise) or Benjamini-Hochberg (BH, for selected pairwise) method when Chi-LRT detected a significant difference (*p* < 0.05). Statistical significance was assumed if *p* < 0.05 (indicated with blue or single asterisk), *p* < 0.01 (green or double asterisk), and *p* < 0.001 (red or triple asterisk).

## Results

### Visualization of nanoscale PI(4,5)P_2_ distribution using SDS-digested freeze-fracture replica labeling

To observe the nanoscale two-dimensional (2D) distribution of PI(4,5)P_2_, SDS-FRL has been used on cultured human fibroblasts, mouse smooth muscle cells, and rat pancreatic exocrine acinar cells (Fujita et al., 2009; Ozato-Sakurai et al., 2011). In this study, we optimized this method for mouse cerebellar tissues to visualize the nanoscale PI(4,5)P_2_ distribution on neuronal cell membranes. We used a recombinant GST-tagged PH domain of PLCδ1 (GST-PH) as a specific probe of PI(4,5)P_2_. To verify the specificity of GST-PH among the PI stereoisomers, we labeled freeze-fracture replicas of liposomes containing either PhdIns or a PI stereoisomer (5 mol%) with GST-PH (50 ng/ml), anti-GST primary antibody, and gold particle-conjugated secondary antibody. The density of immunogold particles was much higher on the replica prepared from PI(4,5)P_2_ liposome compared with those from others (Fig. 1*A*), indicating a high specificity of the probe for PI(4,5)P_2_ among PI stereoisomers (Fig. 1*B*). To assess the labeling efficiency, we labeled PI(4,5)P_2_ on the liposome replicas containing different concentration of PI(4,5)P_2_ (Fig. 1*C*). The particle density increased in a PI(4,5)P_2_ concentration-dependent manner (Fig. 1*D*, left). The labeling efficiency, estimated from the theoretical PI(4,5)P_2_ molecule density (see Materials and Methods), was ∼17% in the range of 0.01–0.1 mol%, while the efficiency drastically dropped to 2–3% for liposomes containing >0.5 mol% PI(4,5)P_2_ (Fig. 1*D*, middle). The mean nearest neighbor distances (NNDs) of the gold particles decreased in a PI(4,5)P_2_-concentration-dependent manner, with a relatively constant NND of ∼20 nm for concentrations above 1 mol% (Fig. 1*D*, right). The mean NNDs for the gold particles were larger than the values calculated from the theoretical PI(4,5)P_2_ molecule densities at all concentrations. Since the diameter of PLCδ1-PH-Ins(1,4,5)P_3_ complex has been reported as ∼6 nm from the crystal structure analysis (Ferguson et al., 1995), the reduction of the labeling efficiency for PI(4,5)P_2_ above 0.5 mol% (mean NND for the theoretical PI(4,5)P_2_ = 8 nm) is probably because of the physical limitation of GST-PH binding to high density PI(4,5)P_2_. Nonetheless, particle density and mean NNDs in the 0.01–5 mol% range are PI(4,5)P_2_ concentration-dependent, indicating that quantitative comparison of PI(4,5)P_2_ distribution pattern is possible.

To evaluate the specificity of GST-PH for PI(4,5)P_2_ labeling on the cell membrane, we introduced a PI(4,5)P_2_ depletion system using rapamycin-inducible heterodimerization proteins (PM-FRB-mRFP-T2A-FKBR-5-ptase; Tóth et al., 2012) into HEK293 cells. In this system, membrane anchor domain and 5-phosphatase (5-ptase) are fused to FRB domain of mTOR (FRB) and its binding partner, FK506 binding protein 12 (FKBR), respectively. Rapamycin induces FRB-FKBR heterodimerization, which rapidly translocates 5-ptase to the cell membrane and converts PI(4,5)P_2_ to PI(4)P (Fig. 1*E*). By the transfection of the plasmid to HEK293 cells, ∼50% of the cells on the dish showed mRFP fluorescence indicating the transfection of the system (Fig. 1*F*). Cells collected by trypsin treatment and centrifugation were applied with rapamycin or vehicle (DMSO), then frozen under high pressure and labeled with PI(4,5)P_2_ by SDS-FRL. We labeled RFP with larger gold particles to identify the transfected cells. Although the particle density for RFP was very low because most of the membrane-anchored PM-FRB-mRFP was probably removed by SDS-digestion, we could identify RFP-positive and negative cells from the difference in the RFP-labeling particle density (RFP-positive: 0.44 ± 0.06 particles/µm^2^, *n* = 10 cells; RFP-negative: 0.07 ± 0.02 particles/µm^2^, *n* = 9 cells; Welch's *t* test: *p* < 0.001). PI(4,5)P_2_-labeling gold particles (PI(4,5)P_2_ particles) were distributed on the cytoplasmic side (P-face) of HEK293 cells (Fig. 1*G*) and the particle density in the transfected cells applied with rapamycin was significantly lower than in other conditions (multiple pairwise comparisons with Tukey adjustment (Tukey): *p* < 0.001; Fig. 1*H*). The PI(4,5)P_2_ molecule density of the transfected/DMSO-applied (control) or/rapamycin-applied (Rap) cells can be estimated to be 4076 and 343 molecules/µm^2^, respectively, based on the gold particle densities (control = 203.8 ± 47.3 particles/µm^2^, Rap = 58.3 ± 13.6 particles/µm^2^; Fig. 1*H*) and the labeling efficiencies estimated in Figure 1*D* (control = 5%, Rap = 17%), indicating that PI(4,5)P_2_ density was reduced ∼92% by the application of rapamycin to the transfected cells. These results demonstrate the specificity of the PI(4,5)P_2_ labeling using SDS-FRL.

To investigate whether PI(4,5)P_2_ is clustered on HEK293 cell membranes, we performed Monte-Carlo random simulations and compared the NNDs between the observed and simulated PI(4,5)P_2_ particles from all images (Szoboszlay et al., 2017). The mean NND of the observed PI(4,5)P_2_ particle distribution was significantly smaller than that of the simulated particle distribution (*n* = 49 images from four cells; observed: 27.8 ± 0.57 nm, simulation: 33.2 ± 0.68 nm; likelihood ratio χ^2^ test (Chi-LRT): *p* < 0.001; Fig. 1*I*), indicating that PI(4,5)P_2_ forms clusters, rather than randomly distributed, on the HEK293 cell membranes.

### Nanoscale PI(4,5)P_2_ distribution on replica preparations of mouse brain tissues

**Figure 2.**
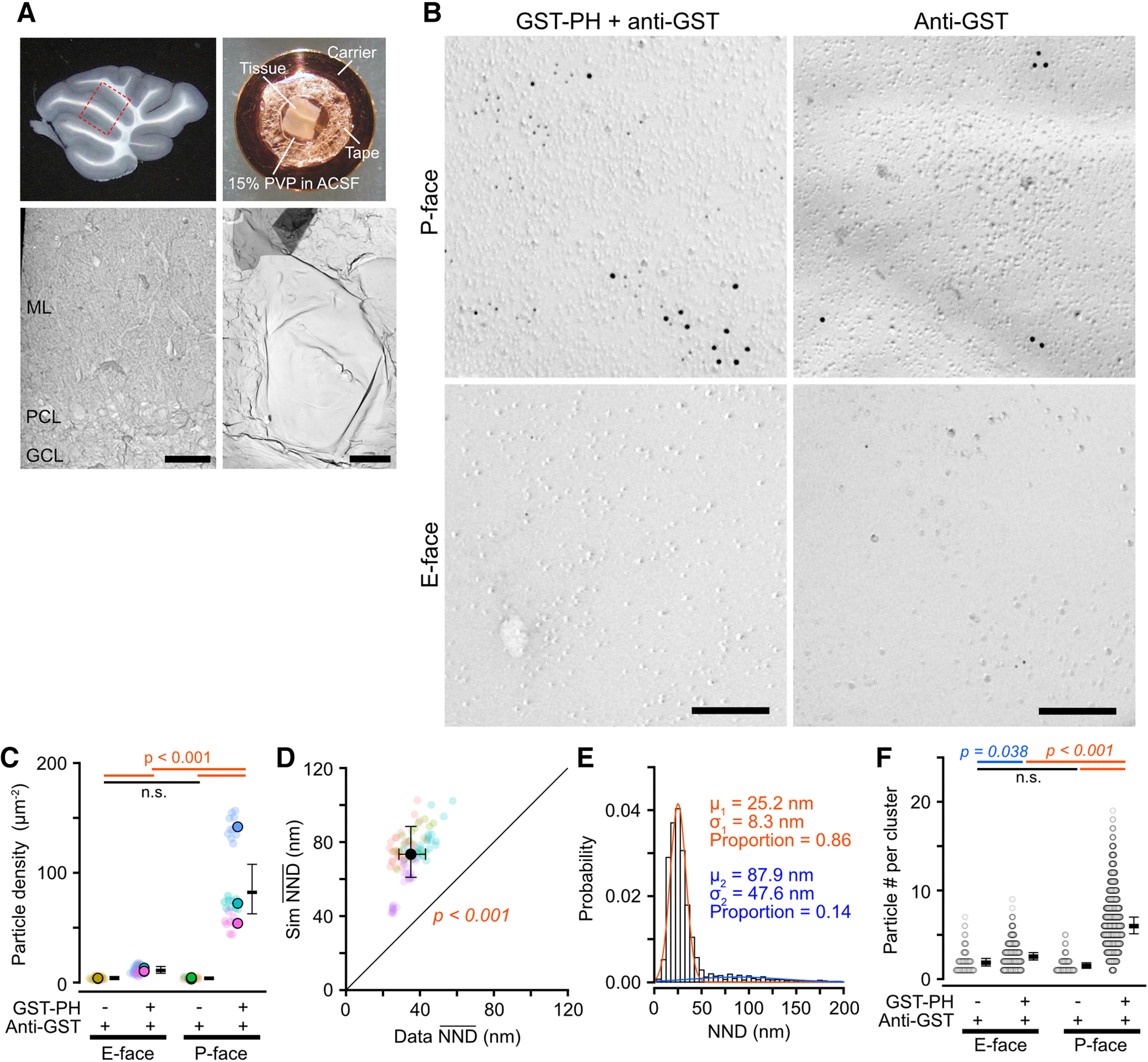
SDS-FRL of PI(4,5)P2 on somatic membranes of Purkinje cells in mouse cerebellum. ***A***, Acute cerebellar slice preparation for high-pressure freezing (HPF) and replica preparation. Left-top, An acute sagittal slice of the mouse cerebellum. The dashed line indicates the trimmed region for HPF. Right-top, A trimmed cerebellar slice on a copper carrier with double-sided tape for HPF. Left-bottom, Low-magnification TEM image of the mouse cerebellar replica containing granule cell layer (GCL), Purkinje cell layer (PCL), and molecular layer (ML). Scale bar = 20 µm. Right-bottom, Example TEM image of the somatic membrane of a PC. Scale bar = 5 µm. ***B***, Example TEM image of 5-nm gold particle labeling for PI(4,5)P_2_ with (left) or without (right) GST-PH on P- (top) and E-face (bottom) of the PC somatic membranes of cerebellar PC. Ca_V_2.1 was co-labeled with 12-nm gold particles. Scale bar = 200 nm. ***C***, Statistical comparison of the PI(4,5)P_2_ particle density on the E-face and P-face of the PC somatic membranes. Closed and transparent circles indicate the means of the PI(4,5)P_2_ particle density in each cell and each image, respectively, with colors indicating different cells. Black horizontal bars and error bars indicate the emmeans and 95% CIs of the density, respectively. The PI(4,5)P_2_ density was significantly higher on the P-face than on the E-face of the PC somatic membranes (P-face: 51.2 ± 8.5 particles/µm^2^, E-face: 8.0 ± 1.2 particles/µm^2^, *n* = 213 images/12 cells/4 mice, *p* < 0.001, Chi-LRT). ***D***, Comparison of NND between real (Data NND, *x*-axis) and simulated (Sim NND, *y*-axis) PI(4,5)P_2_ particles on PC somatic membranes. Data-NNDs are significantly smaller than Sim NNDs (Data: 35.1 ± 3.6 nm, Sim: 73.3 ± 7.0 nm, *n* = 206 images/11 cells/4 mice, *p* < 0.001, Chi-LRT). ***E***, Distribution of NNDs of the PI(4,5)P_2_ particles obtained from a single PC somatic membrane (*n* = 2,929 particles). Red and blue lines indicate the distinct components of the NND distribution estimated from the Gaussian mixture modeling. µ, means; σ, standard deviation. ***F***, Statistical comparison of the number of 5-nm gold particles per cluster on the PC somatic membranes. The particle clusters were detected using Ward Linkage hierarchical clustering method. The linkage distance threshold was set as 50 nm, which is nearby the maximum distance for cluster detection on the PC somatic membrane using DBSCAN (Fig. 3; Materials and Methods). The particle number per cluster without GST-PH was significantly lower than that with GST-PH in both faces (multiple comparisons with BH method), suggesting that the particle clusters observed on the somatic membranes are mainly due to clustering of PI(4,5)P_2_ but not to nonspecific aggregation of the primary and secondary antibodies. n.s., not significant.

To visualize the 2D distribution of PI(4,5)P_2_ on neuronal cell membranes, we labeled PI(4,5)P_2_ in the mouse cerebellum using SDS-FRL with GST-PH. Acute slices were prepared at physiological temperature to maintain the neuronal condition as intact as possible (Eguchi et al., 2020), and then frozen under high pressure with 15% PVP as a cryoprotectant (Fig. 2*A*) to minimize damage caused by ice crystals during freezing (Borges-Merjane et al., 2020). After the SDS-digestion to remove cytosolic proteins and extracellular matrix, cerebellar replicas were sequentially incubated with GST-PH, primary antibodies for GST, and gold particle-conjugated secondary antibodies. Ca_V_2.1 was co-labeled using an antibody against its intracellular domain (Althof et al., 2015), giving specific labeling on the P-face of neuronal cell membranes. Immunogold particles labeling PI(4,5)P_2_ with the GST antibody (PI(4,5)P_2_ particles) showed significantly higher density on the P-face of the somatic membrane of Purkinje cells (PCs) than on the extracellular leaflets (E-face; Chi-LRT: *p* < 0.001; Fig. 2*B*,*C*), indicating the dominance of PI(4,5)P_2_ distribution on the cytoplasmic leaflet of the cell membranes. The particle density without GST-PH was significantly lower on both E- and P-face of the PC somatic membranes than that with GST-PH (Tukey: *p* < 0.001; Fig. 2*B*,*C*), indicating that even the low-density PI(4,5)P_2_ labeling on the E-face is ascribable to GST-PH binding.

To investigate whether PI(4,5)P_2_ is clustered or randomly distributed on somatic membranes of PCs, we compared the NNDs between the observed and randomly distributed PI(4,5)P_2_ particles obtained from the Monte Carlo simulation. The mean NNDs of the observed PI(4,5)P_2_ particles were around half of the simulated one (Chi-LRT: *p* < 0.001; Fig. 2*D*), indicating clustering of PI(4,5)P_2_ on the membrane. The distribution of NNDs between PI(4,5)P_2_ particles obtained from a PC somatic membrane (Fig. 2*E*) is highly right-skewed (skewness = 3.55). Fitting this distribution with Gaussian mixture modeling shows that the NNDs between PI(4,5)P_2_ particles are produced from a mixture of short and long NND populations. These results indicate that the PI(4,5)P_2_ particles on the PC soma has two distinct distribution patterns, clustered and scattered, and that most PI(4,5)P_2_ particles constitute clusters (Fig. 2*E*). The PI(4,5)P_2_ particle clusters were also observed on the E-face of the somatic membrane. The number of particles in a cluster was significantly lower without GST-PH than that with GST-PH on both E- and P-face (Fig. 2*F*). These results indicate that PI(4,5)P_2_ forms clusters on both the outer and inner leaflets of the somatic membranes.

### PI(4,5)P_2_ distribution on somatodendritic compartments of PCs

**Figure 3.**
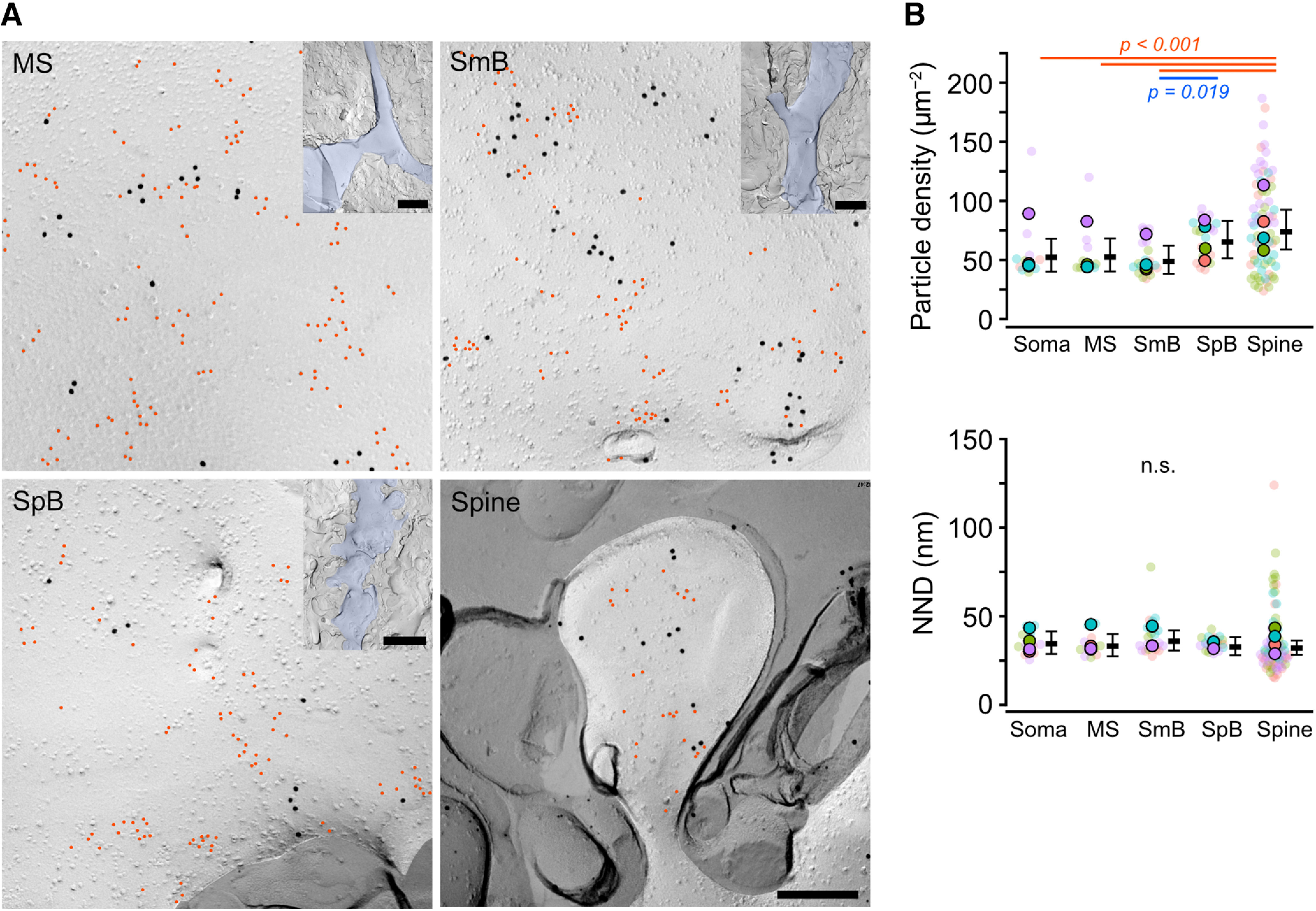
PI(4,5)P_2_ particle distribution on somatodendritic membranes of PCs. ***A***, Example images of PI(4,5)P_2_ labeling (red) with Ca_V_2.1 (black) on the different compartments of PC dendritic membranes: main shaft (MS; top-left), smooth branchlet (SmB; top-right), spiny branchlet (SpB; bottom-left), and spine (bottom-right). Scale bar = 200 nm. Inset, Low-magnification images of MS, SmB, and SpB, respectively, indicated with blue. Red indicates PI(4,5)P_2_ particles. Scale bars = 2 µm (main panels) and 5 µm (insets). ***B***, Comparison of the PI(4,5)P_2_ particle density (top) and NNDs (bottom) in the somatodendritic membrane compartments of PCs. Closed and transparent circles indicate the mean values in each animal and cell, respectively, with colors indicating different animals. Black horizontal bars and error bars indicate the emmeans and 95% CIs, respectively. The PI(4,5)P_2_ density gradually increased from soma and proximal dendrites to distal dendritic components (top, *n* = 611 images/134 components/4 mice, *p* < 0.001, Chi-LRT), whereas no significant difference (n.s.) in NNDs was detected between these compartments (bottom, *n* = 67,380 values/134 components/4 mice, *p* = 0.49, Chi-LRT). See also Table 2.

We next investigate whether the distribution of PI(4,5)P_2_ differs among somatodendritic compartments of PCs: somata, main shafts (MS), smooth branchlets (SmB), spiny branchlets (SpB), and spines. EM pictures of each dendritic component were taken in the molecular layer (ML) divided into proximal (MS), intermediate (SmB) and distal one-third (SpB, spines) of the ML. Gold particles for PI(4,5)P_2_ were observed throughout all the dendritic compartments (Fig. 3*A*). The density of PI(4,5)P_2_ particles was found to increase gradually from the proximal to the distal dendrites (Chi-LRT: *p* < 0.001; Fig. 3*B* top; Table 2): spine membranes showed a ∼1.5 times higher density of PI(4,5)P_2_ particles than somatic, MS, and SmB membranes (Tukey: *p* < 0.001), whereas no significant difference compared with SpB membranes was detected (Tukey: *p* = 0.43). This result indicates the heterogeneous distribution of PI(4,5)P_2_ between the somatodendritic compartments of PCs. Mean NNDs of PI(4,5)P_2_ particles were not significantly different between the compartments (Chi-LRT: *p* = 0.55; Fig. 3*B* bottom; Table 2), indicating that the local concentration of PI(4,5)P_2_ does not differ between different somatodendritic compartments of PCs.

**Table 2.** PI(4,5)P_2_ distribution in somatodendritic membrane compartments of PCs in mouse cerebellum

			95% CI		*p*-value/*z*-score				
Compartment	Mean	SEM	Lower	Upper	Spine	SpB	SmB	MS	Soma
Particle density (µm^2^)									
Soma	52.3	7.0	40.2	68.1	<0.001	0.341	0.949	1.000	—
MS	52.3	7.1	40.2	68.2	<0.001	0.346	0.949	—	−0.002
SmB	48.7	6.0	38.2	62.1	<0.001	0.019	—	0.732	0.732
SpB	62.7	7.8	49.2	80.0	0.149	—	−3.059	−1.848	−1.858
Spine	73.7	8.6	58.6	92.5	—	−2.287	−5.882	−3.917	−3.917
NND (nm)									
Soma	34.3	2.3	30.2	39.0	0.877	1.000	0.871	0.992	—
MS	33.5	2.3	29.3	38.4	0.983	0.994	0.662	—	0.444
SmB	35.9	2.3	31.6	40.8	0.419	0.755	—	−1.347	−0.964
SpB	34.2	2.1	30.3	38.6	0.873	—	1.195	−0.416	0.089
Spine	32.4	2.4	28.0	37.6	—	0.960	1.724	0.544	0.950

MS, main shaft; SmB, smooth branchlet; SpB, spiny branchlet; mean and SEM, marginal means and standard error of means estimated by generalized mixed-effects models; CI, confidential interval; *p*-value, *p*-values obtained using multiple comparison with Tukey method.

### Clustering of PI(4,5)P_2_ on somatodendritic compartments of PCs

**Figure 4.**
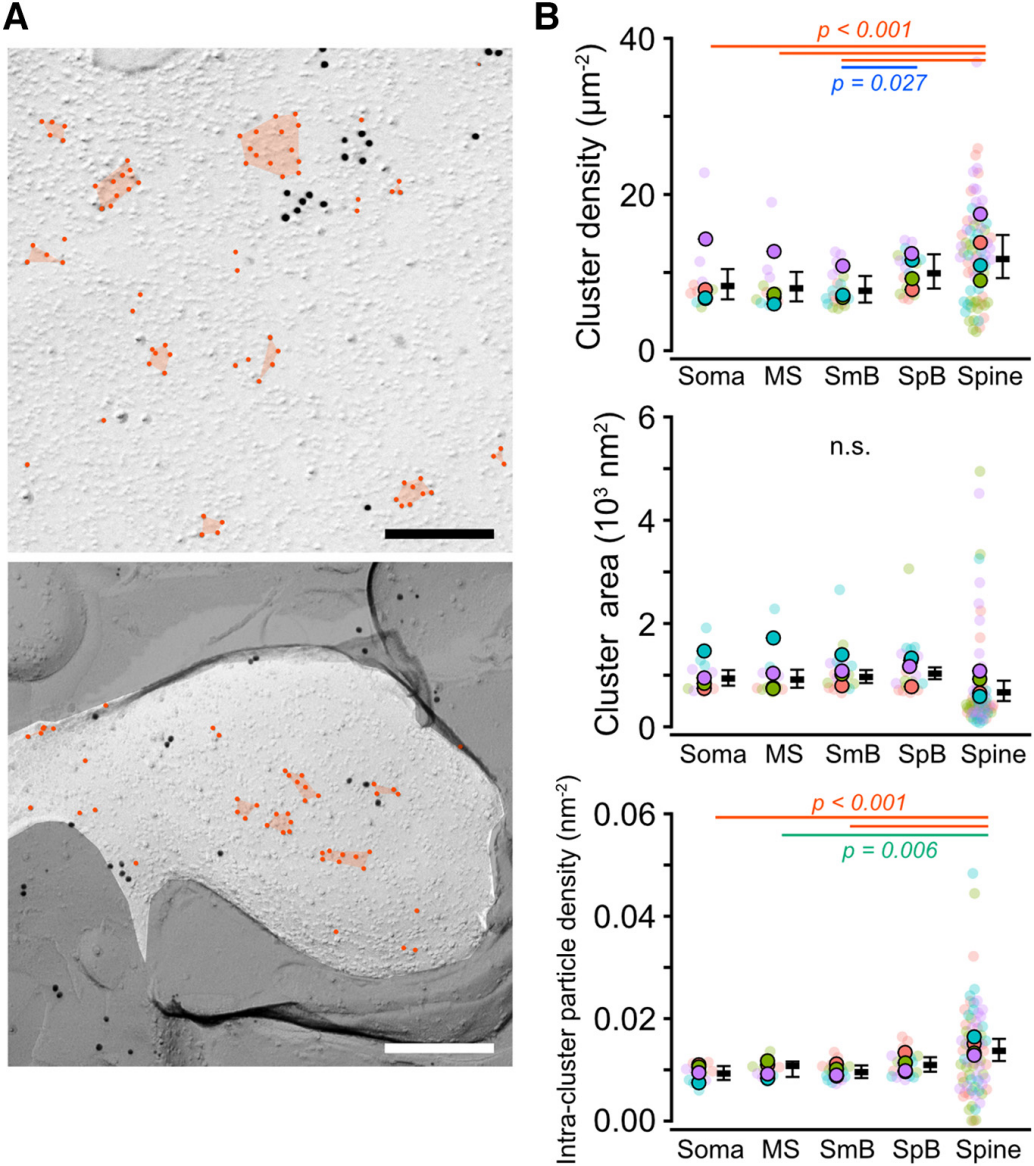
PI(4,5)P_2_ clustering on the somatodendritic membranes of PCs. ***A***, Example images of the PI(4,5)P_2_ clusters on the somatic (top) and spine (bottom) membrane of PCs. Red circles and polygons indicate PI(4,5)P_2_ particles and clusters detected by the DBSCAN algorithm, respectively. Scale bars = 200 nm. ***B***, Quantitative analysis of the PI(4,5)P_2_ clusters on the somatodendritic compartments of PCs. Closed and transparent circles indicate the mean values in each animal and cell, respectively, with colors indicating different animals. Black horizontal bars and error bars indicate the emmeans and 95% CIs, respectively. Top, The number of clusters per area (cluster density, *n* = 611 values/134 components/4 mice). Middle, The cluster area (10,479 clusters/124 components/4 mice). Bottom, The intracluster particle density (10,343 clusters/134 components/4 mice). The cluster density and the intracluster particle density are significantly higher in spine membranes compared to the somatic and proximal dendritic membranes (*p* < 0.001, Chi-LRT), whereas there is no significant difference (n.s.) in the cluster area (*p* = 0.17, Chi-LRT). See also Table 3.

The heterogeneous distribution of PI(4,5)P_2_ between somatodendritic compartments could be caused by the difference in the number, area, or density of the PI(4,5)P_2_ particle clusters because the PI(4,5)P_2_ particles on the dendritic membranes formed clusters as seen on the somatic membranes. To test these possibilities, we compared the PI(4,5)P_2_ cluster profiles (cluster area, particle number in cluster, and intracluster density) between all compartments of somatodendritic membranes. We detected the clusters using the Density-Based Spatial Clustering of Applications with Noise (DBSCAN) algorithm (Szoboszlay et al., 2017). The DBSCAN detected variable sizes of PI(4,5)P_2_ particle clusters on the somatodendritic membranes (Fig. 4*A*). The density of PI(4,5)P_2_ clusters on the PC cell membranes gradually increased from the proximal to the distal compartments (Chi-LRT: *p* < 0.001; Fig. 4*B*, top; Table 3); spine membranes showed a higher density of PI(4,5)P_2_ clusters than somatic, MS, and SmB membranes (Tukey: *p* < 0.001), whereas no significant difference with SpB membranes was detected (Tukey: *p* = 0.21). The cluster area was not significantly different between all compartments of somatodendritic membranes (Chi-LRT: *p* = 0.10; Fig. 4*B*, middle; Table 3), whereas intracluster PI(4,5)P_2_ particle density in spines was significantly higher than those in other compartments (Chi-LRT: *p* < 0.001; Fig. 4*B*, bottom; Table 3). These results suggest that the proximo-distal gradient of PI(4,5)P_2_ along the somatodendritic compartments of PC membranes is ascribable to the differences in the number of PI(4,5)P_2_ clusters and intracluster PI(4,5)P_2_ density, rather than the cluster size, between compartments.

**Table 3.** PI(4,5)P_2_ cluster parameters in somatodendritic membrane compartments of PCs in mouse cerebellum

			95% CI		*p*-value/*z*-score				
Compartment	Mean	SEM	Lower	Upper	Spine	SpB	SmB	MS	Soma
Cluster density (µm^−2^)									
Soma	8.28	1.00	6.55	10.5	<0.001	0.483	0.859	0.990	—
MS	7.95	0.96	6.27	10.1	<0.001	0.221	0.991	—	0.467
SmB	7.67	0.88	6.13	9.60	<0.001	0.027	—	0.455	0.993
SpB	9.41	1.08	7.51	11.8	0.054	—	−2.943	−2.098	−1.622
Spine	11.7	1.42	9.22	14.8	—	−2.701	−5.321	−4.349	−3.945
Cluster area (nm^2^)									
Soma	936	76	798	1098	0.265	0.960	0.999	1.000	—
MS	914	88	757	1105	0.385	0.927	0.991	—	0.185
SmB	965	64	847	1099	0.155	0.992	—	−0.457	−0.288
SpB	1006	68	882	1148	0.085	—	−0.443	−0.812	−0.684
Spine	668	99	500	892	—	2.524	2.270	1.780	2.003
Intracluster particle density (nm^−2^)									
Soma	0.0093	0.0007	0.0080	0.0108	<0.001	0.244	0.996	0.910	—
MS	0.0100	0.0008	0.0086	0.0117	0.006	0.826	0.977	—	−0.865
SmB	0.0096	0.0007	0.0084	0.0109	<0.001	0.319	—	0.588	−0.376
SpB	0.0109	0.0008	0.0095	0.0125	0.055	—	−1.896	−1.063	−2.047
Spine	0.0137	0.0011	0.0117	0.0160	—	−2.693	−4.370	−3.394	−4.308

MS, main shaft; SmB, smooth branchlet; SpB, spiny branchlet; mean and SEM, marginal means and standard error of means estimated by generalized mixed-effects models; CI, confidential interval; *p*-value, *p*-values obtained using multiple comparison with Tukey method.

### PI(4,5)P_2_ distribution on membrane compartments of MLIs and GCs

**Figure 5.**
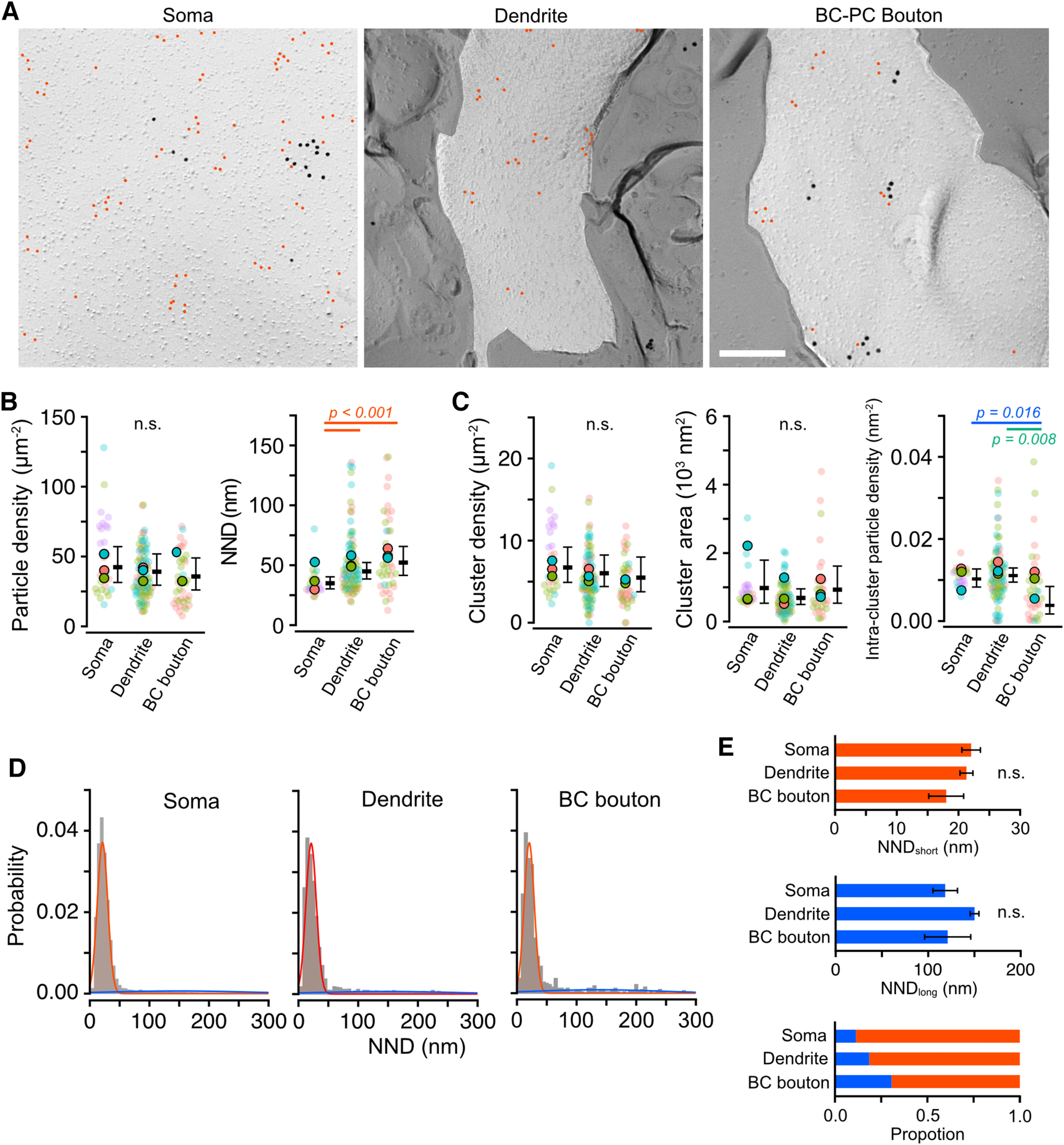
PI(4,5)P_2_ particle distribution in molecular layer interneuron (MLI) membranes. ***A***, Example images obtained from different compartments of MLI membranes: somatic (left), dendrites (middle), and basket cell (BC) presynaptic bouton on PC soma (right). Red indicates PI(4,5)P_2_ particles. Scale bar = 200 nm. ***B***, Comparison of the PI(4,5)P_2_ particle density (left) and NNDs (right) in different membrane compartments of MLIs. Closed and transparent circles indicate the mean values in each animal and cell, respectively, with colors indicating different animals. Black horizontal bars and error bars indicate the emmeans and 95% CIs, respectively. NNDs in dendritic and BC bouton membranes are significantly larger than that in somatic membranes (*n* = 24,012 values/152 components/4 mice, *p* < 0.001, Chi-LRT), whereas there is no significant difference (n.s.) in the density between the compartments (*n* = 230 images/153 components/4 mice, *p* = 0.46, Chi-LRT). ***C***, Quantitative analysis of the PI(4,5)P_2_ particle clusters in different MLI membrane compartments. Closed and transparent circles indicate the mean values in each animal and cell, respectively, with colors indicating different animals. Black horizontal bars and error bars indicate the emmeans and 95% CIs, respectively. The intracluster particle density in BC bouton membranes is significantly smaller than the other components (*n* = 3,766 clusters/149 components/4 mice, *p* = 0.03, Chi-LRT), whereas no significant difference was detected in the cluster density (*n* = 230 images/153 components/4 mice, *p* = 0.43, Chi-LRT) and the cluster area (*n* = 3,822 clusters/146 components/4 mice, *p* = 0.12, Chi-LRT) between these compartments. ***D***, Distribution of NNDs of the PI(4,5)P_2_ particles obtained from the somatic (left, *n* = 20,164 particles), dendritic (middle, *n* = 3,175 particles), and BC bouton (right, *n* = 673 particles) membranes of MLIs. Red and blue lines indicate the shorter and longer components of the NND distribution, respectively, estimated from the Gaussian mixture modeling. ***E***, Mean values of shorter (red, top) and longer (blue, middle) components of the NND of PI(4,5)P_2_ particles and their proportion (bottom) on MLI compartments estimated by the Gaussian mixture modeling. Since there are no significant differences in the mean values of both shorter and longer components of the NND (*p* = 0.21 and 0.24, respectively, Chi-LRT), the higher mean NND values in the dendritic and BC bouton membranes compared to the somatic membranes (Fig. 5*B*) are ascribable to larger proportions of the scattered PI(4,5)P_2_ particles in these compartments. See also Table 4.

**Figure 6.**
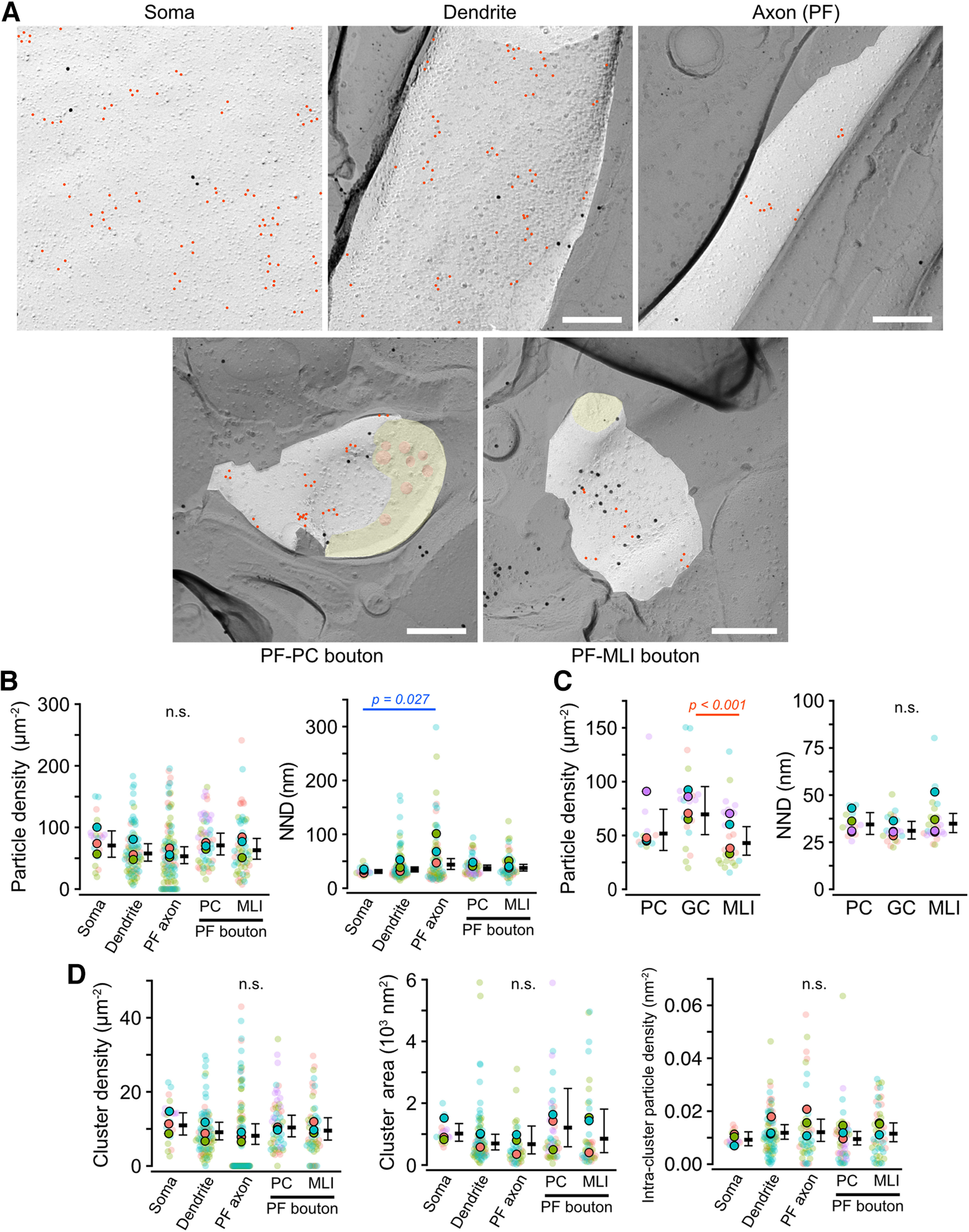
PI(4,5)P_2_ particle distribution in cerebellar granule cell (GC) membranes. ***A***, Example images obtained from the different compartments of GC membranes: soma (top-left), dendrites (top-middle), parallel fiber (PF) axons (top-right), presynaptic PF boutons on the PC spine (PC-PF, bottom-left), and on the MLI dendrite (PF-MLI, bottom-right). Red indicates PI(4,5)P_2_ particles. Yellow and orange in the PF boutons indicate the cross-fracture and synaptic vesicle membranes, respectively. Scale bars = 200 nm. ***B***, Comparison of the PI(4,5)P_2_ particle density (left) and NNDs (right) in the different GC membrane compartments. Closed and transparent circles indicate the mean values in each animal and cell, respectively, with colors indicating different animals. Black horizontal bars and error bars indicate the emmeans and 95% CIs, respectively. NNDs show significant differences between the compartments (*n* = 67,380 values/134 components/4 mice, *p* < 0.001, Chi-LRT), whereas there is no significance (n.s.) in the density (*n* = 376 images/320 components/4 mice, *p* = 0.09, Chi-LRT). ***C***, Comparison of the PI(4,5)P_2_ distribution in somatic membranes between different neuron types in mouse cerebellum. Left, The PI(4,5)P_2_ particle density on the somatic membranes (P-face) of different neuronal cell types in the mouse cerebellum (PC: 51.8 ± 9.6 particles/µm^2^, GC: 69.8 ± 11.3 particles/µm^2^, MLI: 42.9 ± 6.7 particles/µm^2^, *n* = 273 images/57 cells/4 mice, *p* = 0.003, Chi-LRT). Right, NND of the PI(4,5)P_2_ particles on the somatic membranes of different neuronal cell types in the mouse cerebellum (PC: 34.4 ± 3.0 nm, GC: 31.0 ± 2.4 nm, MLI: 34.8 ± 2.6 nm, *n* = 65,607 values/57 cells/4 mice, *p* = 0.12, Chi-LRT). ***D***, Quantitative analysis of the PI(4,5)P_2_ particle clusters in different GC membrane compartments. Closed and transparent circles indicate the mean values in each animal and cell, respectively, with colors indicating different animals. Black horizontal bars and error bars indicate the emmeans and 95% CIs, respectively. There are no significant differences in the cluster density (*n* = 376 images/320 components/4 mice, *p* = 0.37, Chi-LRT), the cluster area (*n* = 4070 clusters/254 components/4 mice, *p* = 0.18, Chi-LRT), and the intracluster particle density (*n* = 4004 clusters/253 compartments/4 mice, *p* = 0.50, Chi-LRT). See also Table 5.

Next, to examine whether the distribution pattern of PI(4,5)P_2_ differs between cerebellar neuron types and whether they show heterogeneous distribution between subcellular compartments as observed in PCs, we visualized the PI(4,5)P_2_ distributions in different compartments of molecular layer interneurons [MLIs; somata, dendrites, presynaptic basket cell boutons on PC somata (BC-PC)] and granule cells [GCs; somata, dendrites, parallel fiber (PF) axons, presynaptic PF boutons with PC spines (PF-PC) or MLI dendrites (PF-MLI)]. Gold particles labeling PI(4,5)P_2_ were distributed throughout all observed subcellular compartments of GCs and MLIs (Figs. 5*A*, 6*A*). In MLIs, the PI(4,5)P_2_ particle density was not significantly different between the membrane compartments, but the mean NNDs were larger in dendritic and presynaptic bouton membranes than in somatic membranes (Fig. 5*B*; Table 4), indicating the higher local PI(4,5)P_2_ density in the somatic membrane. To further investigate differences in local PI(4,5)P_2_ density between the compartments, we analyzed parameters of PI(4,5)P_2_ clusters. The results revealed that the intracluster density of PI(4,5)P_2_ in the bouton membrane was significantly lower than in other compartments, whereas the cluster density and area were similar (Chi-LRT: *p* = 0.43 and 0.12, respectively; Fig. 5*C*; Table 4). Histograms of NNDs between PI(4,5)P_2_ particles were fitted with a Gaussian mixture modeling to obtain the mean NNDs and proportions for the short (clustered) and long (sparse) NND groups, respectively (Fig. 5*D*). The results show no significant difference in the mean NNDs of both groups between the compartments [Chi-LRT: *p* = 0.21 (short) and 0.12 (long); Fig. 5*E*], indicating that the difference in mean NND between the MLI compartments is because of the difference of the proportion of the clustered PI(4,5)P_2_. These results suggest that, in MLI, a higher proportion of PI(4,5)P_2_ forms denser clusters on the somatic membrane compared with dendritic and presynaptic bouton membranes.

**Table 4.** PI(4,5)P_2_ distribution in different membrane compartments of MLIs in mouse cerebellum

			95% CI		*p*-value^†^/*z*-score		
Compartment*	Mean**	SEM	Lower	Upper	BC-PC	Dendrite	Soma
Particle density (µm^−2^)							
Soma	42.4	6.5	31.4	57.1	0.449	0.789	—
Dendrite	39.1	5.6	29.5	51.9	0.649	—	0.656
BC-PC bouton	35.7	5.8	26.1	49.0	—	0.887	1.208
NND (nm)							
Soma	34.9	2.6	30.3	40.3	<0.001	<0.001	—
Dendrite	44.9	3.5	38.5	52.2	0.358	—	−3.612
BC-PC bouton	52.2	6.1	41.5	65.7	—	−1.368	−3.650
Cluster density (µm^−2^)							
Soma	6.73	1.08	4.91	9.21	0.417	0.635	—
Dendrite	6.02	0.96	4.40	8.24	0.791	—	0.908
BC-PC bouton	5.49	1.05	3.77	8.00	—	0.652	1.261
Cluster area (nm^2^)							
Soma	979	303	534	1794	0.994	0.307	—
Dendrite	690	115	498	955	0.667	—	1.468
BC-PC bouton	931	263	535	1619	—	−0.857	0.109
Intracluster particle density (nm^−2^)							
Soma	0.0103	0.0011	0.0083	0.0127	0.008	0.747	
Dendrite	0.0111	0.0009	0.0095	0.0129	0.016		−0.727
BC-PC bouton	0.0038	0.0015	0.0017	0.0084		2.763	2.975

BC, backet cell; PC, Purkinje cell; mean and SEM, marginal means and standard error of means estimated by generalized mixed-effects models; CI, confidential interval; *p*-value, *p*-values obtained using multiple comparison with Tukey method.

In the GC membranes, the density of PI(4,5)P_2_ particles was not significantly different between the compartments (Chi-LRT: *p* = 0.095), though the mean NNDs of PI(4,5)P_2_ particles were slightly but significantly different between somatic and axonal membranes (Tukey: *p* = 0.027; Fig. 6*B*; Table 5). To examine whether the density of PI(4,5)P_2_ differs between neuronal cell types in the cerebellum, we compared the PI(4,5)P_2_ density on the somatic membranes of PCs, GCs, and MLIs. Although the density of PI(4,5)P_2_ particles in the somatic membrane of GCs was significantly higher than that of MLIs (Tukey: *p* < 0.001), the NNDs of the PI(4,5)P_2_ particles on somatic membranes of the cerebellar neurons did not differ between the cell types (Chi-LRT: *p* = 0.12; Fig. 6*C*), indicating a similar local concentration of PI(4,5)P_2_ in these clusters across the examined cerebellar neuronal cell types. The cluster parameters of PI(4,5)P_2_ were also not significantly different between the compartments (Fig. 6*D*; Table 5). These results suggest that PI(4,5)P_2_ in GC membranes shows a similar distribution pattern throughout the observed subcellular compartments.

**Table 5.** PI(4,5)P_2_ distribution in different membrane compartments of GCs in mouse cerebellum

			95% CI		*p*-value/*z*-score				
Compartment	Mean	SEM	Lower	Upper	PF-MLI	PF-PC	PF axon	Dendrite	Soma
Particle density (µm^−2^)									
Soma	68.8	10.8	51.6	94.4	0.961	1.000	0.339	0.658	—
Dendrite	57.7	7.2	45.3	73.6	0.916	0.309	0.916	—	1.353
PF axon	53.2	6.9	41.2	68.7	0.540	0.088	—	0.845	1.860
PF-PC bouton	70.9	8.9	55.3	90.7	0.864	—	−2.510	−1.916	−0.108
PF-MLI bouton	63.0	8.5	48.4	82.1	—	0.980	−1.534	−0.847	0.679
NND (nm)									
Soma	30.8	1.8	27.6	34.5	0.153	0.054	0.027	0.521	—
Dendrite	34.5	2.4	30.2	39.4	0.889	0.848	0.298	—	−1.563
PF axon	43.7	5.1	34.8	54.9	0.775	0.726	—	−1.937	−2.944
PF-PC bouton	37.4	2.6	32.7	42.8	1.000	—	1.243	−1.017	−2.701
PF-MLI bouton	37.5	3.1	31.9	44.2	—	−0.023	1.158	−0.922	−2.274
Cluster density (µm^−2^)									
Soma	10.94	1.51	8.35	14.3	0.887	0.994	0.357	0.541	—
Dendrite	9.11	1.20	7.04	11.8	0.997	0.832	0.937	—	1.534
PF axon	8.14	1.39	5.82	11.4	0.885	0.582	—	0.780	1.828
PF-PC bouton	10.37	1.47	7.86	13.7	0.982	—	−1.470	−1.050	0.414
PF-MLI bouton	9.5	1.51	6.99	13.0	—	0.552	−0.931	−0.347	0.926
Cluster area (nm^2^)									
Soma	1016	144	770	1340	0.990	0.971	0.772	0.053	—
Dendrite	696	127	488	995	0.974	0.340	1.000	—	2.705
PF axon	673	215	360	1260	0.992	0.749	—	0.091	1.164
PF-PC bouton	1208	442	590	2475	0.869	—	−1.205	−1.859	−0.924
PF-MLI bouton	851	327	400	1808	—	0.969	−0.439	−0.610	0.558
Intracluster particle density (nm^−2^)									
Soma	0.0092	0.0009	0.0077	0.0110	0.989	0.991	0.993	0.119	—
Dendrite	0.0123	0.0017	0.0094	0.0160	0.845	0.370	0.703	—	−2.385
PF axon	0.0082	0.0022	0.0048	0.0139	0.976	1.000	—	1.281	0.432
PF-PC bouton	0.0086	0.0014	0.0063	0.0117	0.968	—	−0.163	1.807	0.455
PF-MLI bouton	0.0101	0.0023	0.0065	0.0157	—	−0.642	−0.593	1.023	−0.480

PF, parallel fiber; PC, Purkinje cell; MLI, molecular layer interneuron; mean and SEM, marginal means and standard error of means estimated by generalized mixed-effects models; CI, confidential interval; *p*-value, *p*-values obtained using multiple comparison with Tukey method.

### PI(4,5)P_2_ distribution on synaptic membranes

**Figure 7.**
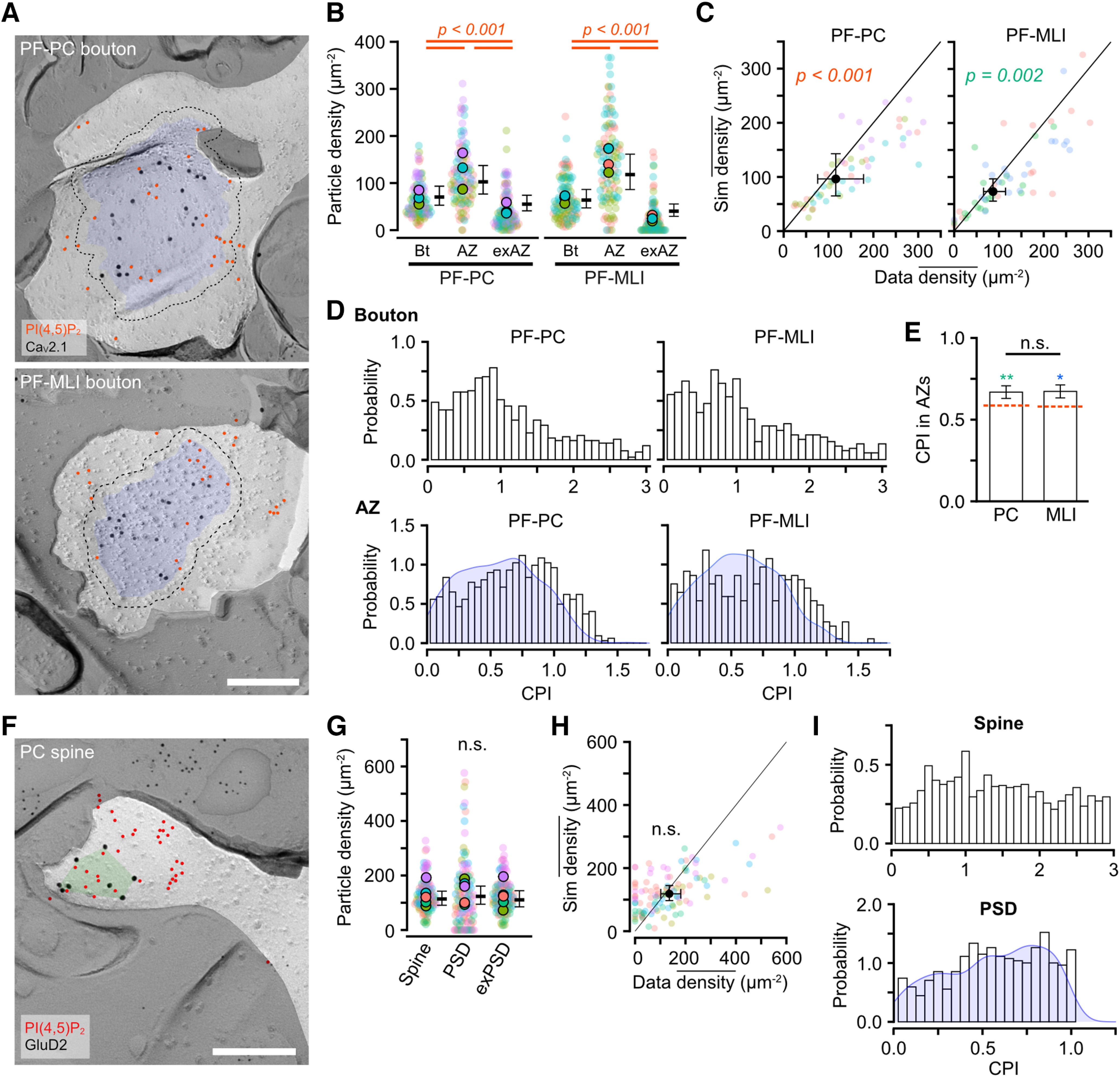
PI(4,5)P_2_ distribution in presynaptic and postsynaptic membranes of PF-PC synapses. ***A***, Example images for PI(4,5)P_2_ particle distribution on the membranes of PF-PC (top) and PF-MLI bouton (bottom). Red and black circles indicate gold particles for PI(4,5)P_2_ and Ca_V_2.1, respectively. The Blue area and dotted line indicate AZ and outer-rim (30 nm from the edge of AZ; Materials and Methods), respectively. Scale bar = 200 nm. ***B***, Beeswarm plot of the PI(4,5)P_2_ density in the whole bouton (Bt), AZs, and extra-AZ region (exAZ) of the PF-PC (left) and PF-MLI (right) bouton membranes. Closed and transparent circles indicate the mean values in each animal and bouton, respectively, with colors indicating different animals. Black horizontal bars and error bars indicate the emmeans and 95% CIs, respectively. The PI(4,5)P_2_ density was significantly higher in AZs than in the whole bouton and the exAZ in both PF-PC and PF-MLI boutons (*n* = 111 boutons/4 mice, *p* < 0.001, Tukey method). ***C***, Comparison of the PI(4,5)P_2_ density in the AZs between real and simulated random distribution on the PF bouton membranes. Transparent circles indicate the mean densities of each AZ with colors indicating different animals. Black circle and error bars indicate the emmeans and 95% CIs, respectively. The density of the real particle distribution was significantly higher than that of the simulated one in both PF-PC (real: 97.2 ± 19.7 particles/µm^2^, sim: 66.7 ± 13.5 particles/µm^2^, *n* = 55 boutons/4 mice, *p* < 0.001, Chi-LRT) and PF-MLI (real 86.7 ± 12.4 particles/µm^2^, sim: 73.2 ± 10.4 particles/µm^2^, *n* = 56 boutons/4 mice, *p* = 0.002, Chi-LRT) AZ membranes. ***D***, Distribution of center-periphery index (CPI) of PI(4,5)P_2_ particles in boutons (top) and AZs (bottom). Blue in the bottom graph indicates the CPI distribution of the simulated particles that are randomly distributed in AZs. ***E***, Comparison of CPIs of the PI(4,5)P_2_ particles in AZs between PF-PC and PF-MLI AZs. There is no significant difference in the CPIs between PF-PC and PF-MLI AZs (PF-PC: 0.67 ± 0.02, PF-MLI: 0.67 ± 0.02, *n* = 107 boutons/4 mice, *p* = 0.999, Chi-LRT). Dashed lines with red show the mean CPIs of the simulated randomly-distributed particles. Asterisks on the bars indicate statistical differences in CPIs between real and simulated particles (PF-PC: 0.59 ± 0.02, PF-MLI: 0.58 ± 0.02, *p < 0.05, **p < 0.01, Tukey method). ***F***, An example image of the PI(4,5)P_2_ particle distribution on the PC spine membrane. Red and black circles indicate PI(4,5)P_2_ and GluD2, respectively. The green area indicates postsynaptic density (PSD) based on the cluster of GluD2. Scale bar = 200 nm. ***G***, Beeswarm plot of the PI(4,5)P_2_ density in the whole spine (spine), PSD, and extra-PSD region (exPSD) of the PC spine membranes. Closed and transparent circles indicate the mean values in each animal and spine, respectively, with colors indicating different animals. Black horizontal bars and error bars indicate the emmeans and 95% CIs, respectively. No significant difference (n.s.) in the density was detected between these compartments (*n* = 108 spines/6 mice, *p* = 0.77, Chi-LRT). ***H***, Comparison of the PI(4,5)P_2_ density in the PSDs between real and simulated random distribution on the PC spine membranes. Transparent circles indicate the mean densities of each PSD with colors indicating different animals. Black circle and error bars indicate the emmeans and 95% CIs, respectively. The density of the real and simulated particle distribution was not significantly different (real: 0.59 ± 0.01, sim: 0.55 ± 0.01, *n* = 108 spines/6 mice, *p* = 0.25, Chi-LRT). ***I***, Distribution of CPIs of PI(4,5)P_2_ particles in spines (top) and PSDs (bottom). Blue in the bottom graph indicates the CPI distribution of the simulated particles that are randomly distributed in PSDs. The CPI of the PI(4,5)P_2_ particles is uniformly distributed in the spines and PSDs, suggesting the random distribution of PI(4,5)P_2_. See also Table 6.

Since many presynaptic proteins related to neurotransmitter release contain PI(4,5)P_2_-binding domains (T.F.J. Martin, 2001), PI(4,5)P_2_ is expected to be localized at the presynaptic active zones (AZs). To address this question, we focused on the PI(4,5)P_2_ particle distribution in the presynaptic membrane of PF (Fig. 7*A*). The PI(4,5)P_2_ particle density was significantly higher in the AZs than in the whole bouton and extra-AZ membranes at both PF-PC and PF-MLI synapses (Tukey: *p* < 0.001; Fig. 7*B*; Table 6). The observed density of PI(4,5)P_2_ particles in AZs was significantly higher than that of randomly distributed PI(4,5)P_2_ particles on the bouton membrane by Monte-Carlo simulation [Chi-LRT: *p* < 0.001 (PF-PC) and *p* = 0.002 (PF-MLI); Fig. 7*C*], suggesting the accumulation of the PI(4,5)P_2_ in the AZ of PF boutons. Next, we examined how the PI(4,5)P_2_ particles are distributed within the AZs with center-periphery index (CPI), where a CPI of 0 indicates that the particle is at the center of gravity, and a CPI of 1 indicates the particle is at the edge of the AZ (Kleindienst et al., 2020). The histogram of CPI in the bouton membranes demonstrates that the particles are distributed at a high density within the AZ and that the distribution probability decreases with distance from the AZ (Fig. 7*D*, top), suggesting the PI(4,5)P_2_ accumulation in the AZ. The CPI distribution within AZs showed higher probability at around CPI = 1.0 compared with that of the random simulation (Fig. 7*D*, bottom), indicating that PI(4,5)P_2_ is preferentially located in the periphery of the AZs. The mean CPI of the PI(4,5)P_2_ particles in AZs was significantly higher than that of randomly distributed particles in both PF-PC and PF-MLI synapses (Chi-LRT: *p* < 0.001; Fig. 7*E*). These results suggest that the AZ, especially its periphery, is enriched in PI(4,5)P_2_, regardless of the postsynaptic cell types.

**Table 6.** PI(4,5)P_2_ particle density in synaptic membranes of mouse cerebellar neurons (particles/µm^2^)

			95% CI		*p*-value/*z*-score		
Compartment	Mean	SEM	Lower	Upper	exAZ	AZ	Bouton
PF-PC boutons							
Bouton	70.2	10.2	52.9	93.3	<0.001	<0.001	—
AZ	102.6	15.2	76.7	137.2	<0.001	—	−5.908
exAZ	55.1	8.3	40.9	74.1	—	6.952	4.018
PF-MLI boutons							
Bouton	63.8	9.9	47.2	86.4	<0.001	<0.001	—
AZ	118.0	18.9	86.2	161.3	<0.001	—	−8.089
exAZ	40.3	6.6	29.2	55.6	—	6.952	5.950
PC spines							
Spine	114.0	12.9	91.0	142	0.749	0.840	—
PSD	124.0	16.5	95.6	161	0.805	—	−0.564
exPSD	111.0	14.8	85.0	144	—	0.628	0.724

PF, parallel fiber; PC, Purkinje cell; MLI, molecular layer interneuron; AZ, active zone; ezAZ, extra AZ; PSD, postsynaptic density; exPSD, extra PSD; mean and SEM, marginal means and standard error of means estimated by generalized mixed-effects models; CI, confidential interval; *p*-value, *p*-values obtained using multiple comparison with Tukey method.

Next, we investigated the PI(4,5)P_2_ distribution in PSD of dendritic spines. The PI(4,5)P_2_ particles were distributed throughout the spine membranes (Fig. 7*F*), and the density in the PSDs was not significantly different compared with the whole spine and extra-PSD membranes (Chi-LRT: *p* = 0.77; Fig. 7*G*; Table 6). The observed PI(4,5)P_2_ particle density in PSDs was not significantly higher than that of the particles randomly distributed in the spine (Chi-LRT: *p* = 0.25; Fig. 7*H*). Furthermore, the CPI of the PI(4,5)P_2_ particles in the spines was uniformly distributed, and the CPI in the PSDs was similar to that of the simulated particles randomly distributed (Fig. 7*I*). These results suggest that PI(4,5)P_2_ is not specifically accumulated in the postsynaptic site of PC dendritic spines.

### The association of ion channels and receptors with PI(4,5)P_2_ on cell membranes of cerebellar neurons

The visualization of PI(4,5)P_2_ allows us to investigate the physical association of ion channels and neurotransmitter receptors with PI(4,5)P_2_, which helps to understand the physiological role of PI(4,5)P_2_ on neuronal functions. We examined whether these membrane proteins associate with PI(4,5)P_2_ on cell membranes by double labeling in mouse cerebellar neurons.

#### The association of Ca_V_2.1 with PI(4,5)P_2_

**Figure 8.**
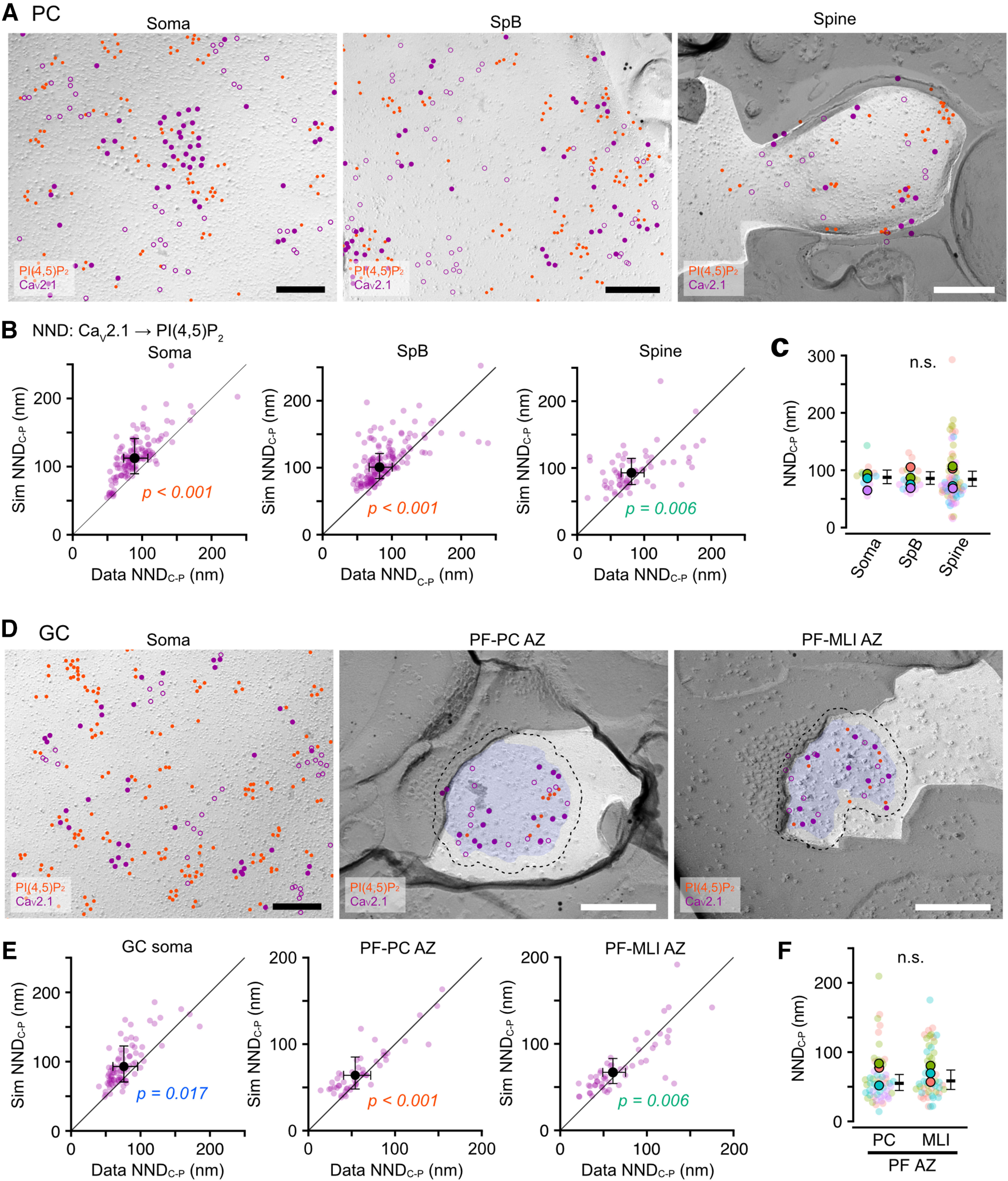
Ubiquitous association of Ca_V_2.1 with PI(4,5)P_2_ on cell membranes of PCs and GCs. ***A***, Example images for co-immunolabeling of PI(4,5)P_2_ and Ca_V_2.1 on somatic (left), SpB (middle), and spine (right) membranes of the PC. Red and purple (closed, open) circles indicate PI(4,5)P_2_ and Ca_V_2.1 (real, fitted-simulated) particles, respectively. Scale bars = 200 nm. ***B***, Comparison of the NNDs from Ca_V_2.1 to PI(4,5)P_2_ particles (NND_C-P_) between real and fitted-simulated Ca_V_2.1 distribution on somatic (left), SpB (middle), and spine (right) membranes of PCs. The NND_C-P_ of the real distribution was significantly smaller than that of the simulated one in somatic (real: 89.7 ± 9.6 nm, sim: 112.9 ± 12.1 nm, *n* = 129 images/14 cells/5 mice, *p* < 0.001, Chi-LRT), SpB (real: 82.3 ± 8.1 nm, sim: 100.5 ± 9.9 nm, *n* = 158 images/20 dendrites/4 mice, *p* < 0.001, Chi-LRT), and spine membrane (real: 80.9 ± 8.7 nm, sim: 92.8 ± 9.9 nm, 69 spines/4 mice, *p* = 0.006, Chi-LRT). ***C***, Comparison of NND_C-P_ between somatodendritic compartments. Closed and transparent circles indicate the mean values in each animal and cell, respectively, with colors indicating different animals. Black horizontal bars and error bars indicate the emmeans and 95% CIs, respectively. There is no significant difference (n.s.) between the PC compartments (*n* = 12,956 values/92 components/5 mice, *p* = 0.70, Chi-LRT). ***D***, Example images for co-immunolabeling of PI(4,5)P_2_ and Ca_V_2.1 on somatic (left) and presynaptic PF-PC (middle) and PF-MLI (right) AZ membranes of the GC. Red and purple (closed, open) circles indicate PI(4,5)P_2_ and Ca_V_2.1 (real, fitted-simulated) particles, respectively. The Blue area and dotted lines indicate AZs and the outer-rim, respectively. Scale bars = 200 nm. ***E***, Comparison of the NND_C-P_ between real and fitted-simulated Ca_V_2.1 distribution on somatic (left), PF-PC AZ (middle), and PF-MLI AZ (right) membranes of GCs. The NND_C-P_ of the real distribution was significantly smaller than that of the simulated one in somatic (real: 76.0 ± 9.0 nm, sim: 93.0 ± 13.1 nm, *n* = 81 images/25 cells/5 mice, *p* = 0.017, Chi-LRT), PF-PC AZ (real: 54.0 ± 7.9 nm, sim: 64.0 ± 9.3 nm, *n* = 54 AZs/4 mice, *p* < 0.001, Chi-LRT), and PF-MLI AZ membrane (real: 60.8 ± 6.7 nm, sim: 67.0 ± 7.3 nm, 52 AZs/4 mice, *p* = 0.006, Chi-LRT). ***F***, Comparison of NND_C-P_ between presynaptic AZs of PF-PC and PF-MLI synapses. Closed and transparent circles indicate the mean values in each animal and AZ, respectively, with colors indicating different animals. Black horizontal bars and error bars indicate the emmeans and 95% CIs, respectively. There is no significant difference in NND_C-P_ between the AZs (*n* = 1110 values/68 AZs/4 mice, *p* = 0.60, Chi-LRT).

**Figure 9.**
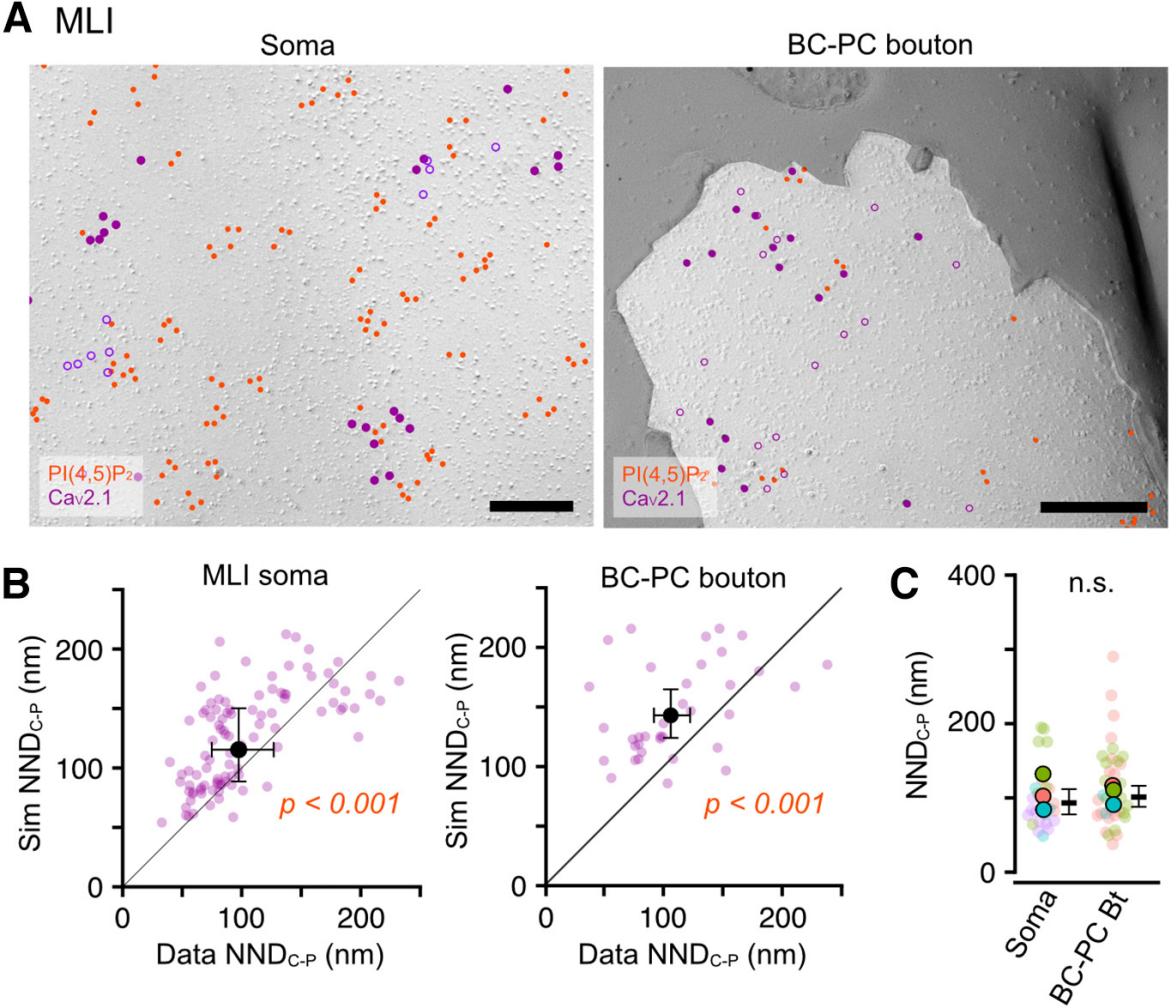
Association of Ca_V_2.1 with PI(4,5)P_2_ on cell membranes of MLIs. ***A***, Example images for co-immunolabeling of PI(4,5)P_2_ and Ca_V_2.1 on somatic (left) and basket cell (BC)-PC bouton (right) membranes of the MLI. Red and purple (closed, open) circles indicate PI(4,5)P_2_ and Ca_V_2.1 (real, fitted-simulated) particles, respectively. The P-face of BC-PC boutons were identified based on Ca_V_2.1 clusters and the surrounding E-face of the PC somatic membranes with Ca_V_2.1 clusters. Scale bars = 200 nm. ***B***, Comparison of the NND_C-P_ between real and fitted-simulated Ca_V_2.1 distribution on somatic (left) and BC-PC bouton (right) membranes of MLIs. The NND_C-P_ of the real distribution was significantly smaller than that of the simulated one in somatic (real: 97.5 ± 13.1 nm, sim: 115.4 ± 15.5 nm, *n* = 102 images/26 cells/4 mice, *p* < 0.001, Chi-LRT) and BC-PC bouton membrane (real: 106.0 ± 7.7 nm, sim: 143.0 ± 10.4 nm, 37 boutons/3 mice, *p* = 0.006, Chi-LRT). ***C***, Comparison of NND_C-P_ between somatic and bouton membranes. Closed and transparent circles indicate the mean values in each animal and cell, respectively, with colors indicating different animals. Black horizontal bars and error bars indicate the emmeans and 95% CIs, respectively. No significant difference (n.s.) was shown between these compartments (*n* = 2854 values/63 components/4 mice, *p* = 0.29, Chi-LRT).

Ca_V_2.1 is an α-subunit of P/Q-type voltage-gated calcium channels and is regulated by PI(4,5)P_2_ directly or indirectly through Ca_V_ β-subunit (Suh and Hille, 2008; Suh et al., 2012). Here, we examined whether Ca_V_2.1 is associated with PI(4,5)P_2_ in the neuronal membranes by co-immunolabeling of Ca_V_2.1 with the PI(4,5)P_2_ labeling. To assess the association, we compared NNDs from Ca_V_2.1 to PI(4,5)P_2_ particles (NND_C-P_) between observed and simulated Ca_V_2.1 particles. To reproduce the Ca_V_2.1 clustering in the simulation, we performed a fitted simulation of Ca_V_2.1 particle distribution (Materials and Methods; Luján et al., 2018b; Kleindienst et al., 2020). In PCs, Ca_V_2.1 was broadly expressed in somatodendritic membranes (Fig. 8*A*), as previously reported (Indriati et al., 2013). The real values of NND_C-P_ in somatic, SpB, and spine membranes were significantly shorter than the values obtained with the simulated Ca_V_2.1 distribution (Fig. 8*B*), indicating the association of Ca_V_2.1 with PI(4,5)P_2_ on the somatodendritic membranes. The mean NND_C-P_ was not significantly different between the compartments of PC membranes (Fig. 8*C*). In GCs, Ca_V_2.1 was highly expressed in somatic and presynaptic AZ membranes (Fig. 8*D*). The comparison of NND_C-P_ between observed and simulated Ca_V_2.1 particles showed significant differences in the somatic and PF-PC/MLI AZ membranes (Fig. 8*E*), indicating the association of Ca_V_2.1 with PI(4,5)P_2_. The mean NND_C-P_ values in PF-PC and PF-MLI AZs were not significantly different (Fig. 8*F*). In MLI, Ca_V_2.1 was expressed in somatic and presynaptic basket cell (BC)-PC bouton membranes (Fig. 9*A*). The NND_C-P_ in somatic and BC-PC bouton membranes was significantly shorter than the simulated one (Fig. 9*B*). The mean NND_C-P_ was not significantly different between the somatic and BC-PC bouton membranes of the MLIs (Fig. 9*C*). These results suggest the ubiquitous association of Ca_V_2.1 with PI(4,5)P_2_ in neuronal cell membranes across various cell types and their compartments.

#### The association of GIRK3 channels with PI(4,5)P_2_

**Figure 10.**
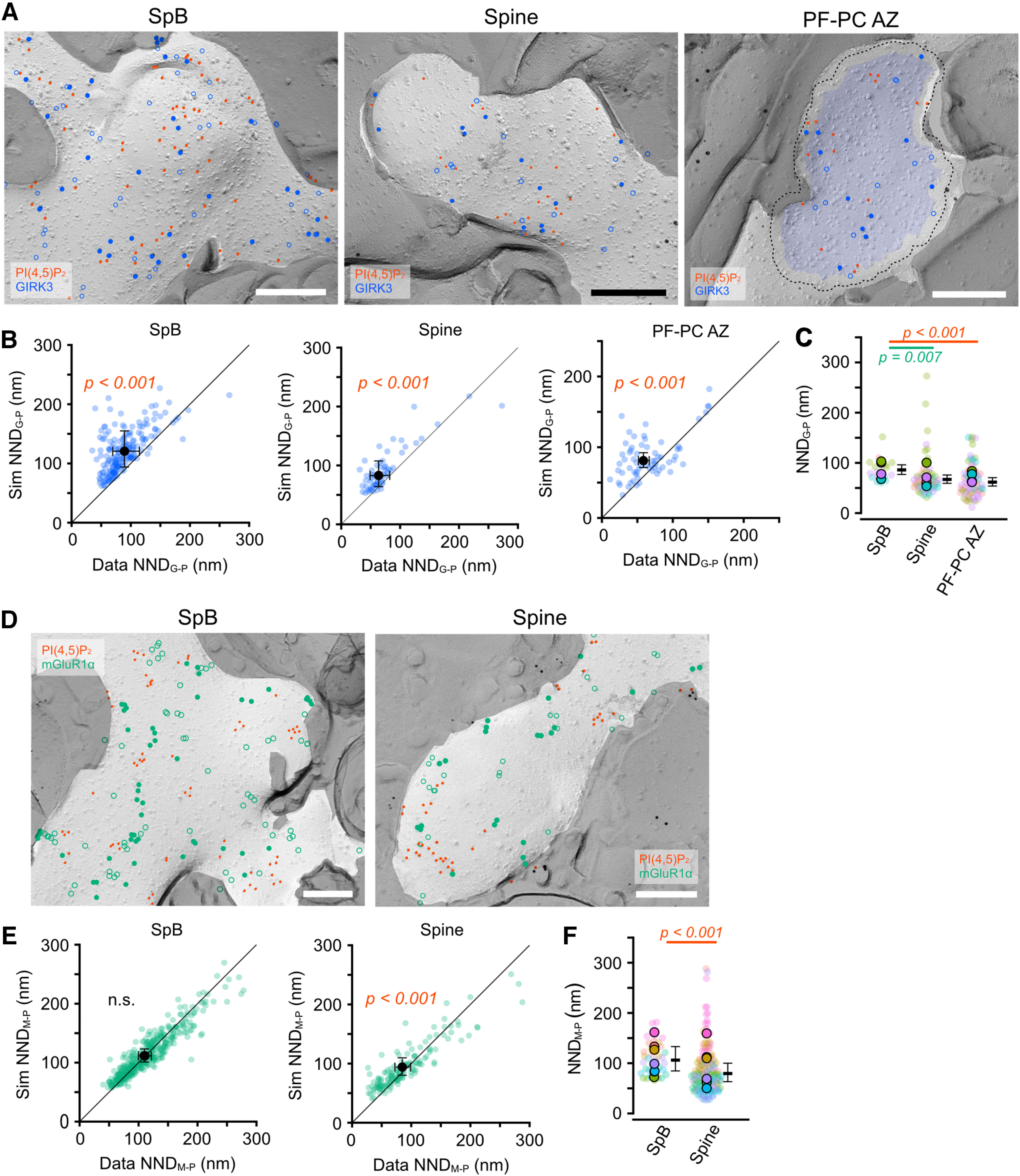
Association of GIRK3 channels and mGluR1α receptors with PI(4,5)P_2_ on cell membranes of cerebellar neurons. ***A***, Example images for co-labeling of PI(4,5)P_2_ and GIRK3 channels on PC SpB (left), PC spine (middle), and PF-PC AZ (right) membranes. Red and blue (closed, open) circles indicate PI(4,5)P_2_ and GIRK3 (real, fitted-simulated) particles, respectively. The Blue area and a dotted line on the left indicate the AZ and the outer-rim, respectively. Scale bars = 200 nm. ***B***, Comparison of the NNDs from GIRK3 to PI(4,5)P_2_ particles (NND_G-P_) between real and fitted-simulated GIRK3 distribution on PC SpB (left), PC spine (middle), and PF-PC AZ (right) membranes. The NND_G-P_ of the real distribution was significantly smaller than that of the simulated one in SpB (real: 89.3 ± 11.4 nm, sim: 120.7 ± 15.4 nm, *n* = 88 images/19 dendrites/5 mice, *p* < 0.001, Chi-LRT), spine (real: 63.6 ± 8.4 nm, sim: 83.0 ± 11.0 nm, *n* = 57 spines/4 mice, *p* < 0.001, Chi-LRT), and PF-PC AZ membrane (real: 58.6 ± 3.9 nm, sim: 81.1 ± 5.3 nm, 65 AZs/4 mice, *p* < 0.001, Chi-LRT). ***C***, Comparison of NND_G-P_ between the postsynaptic and presynaptic compartments of cerebellar neurons. Closed and transparent circles indicate the mean values in each animal and cell, respectively, with colors indicating different animals. Black horizontal bars and error bars indicate the emmeans and 95% CIs, respectively. The NND_G-P_ was significantly shorter in the spine and AZ membranes than in the SpB membrane (4730 values/90 compartments/4 mice, *p* < 0.001, Chi-LRT). ***D***, Example images for co-labeling of PI(4,5)P_2_ and mGluR1α receptors on PC SpB (left) and spine (right) membranes. Red and green (closed, open) circles indicate PI(4,5)P_2_ and mGluR1α (real, fitted-simulated) particles, respectively. Scale bars = 200 nm. ***E***, Comparison of the NNDs from mGluR1α to PI(4,5)P_2_ particles (NND_M-P_) between real and fitted-simulated mGluR1α distribution on PC SpB (left) and spine (right) membranes. The NND_M-P_ of the real distribution was significantly smaller than that of the simulated one in the spine membranes (real: 81.8 ± 18.4 nm, sim: 91.2 ± 20.6 nm, *n* = 140 spines/7 mice, *p* < 0.001, Chi-LRT), but not in the SpB membrane (*n* = 147 images/35 dendrites/7 mice, *p* = 0.36, Chi-LRT). ***F***, Comparison of NND_M-P_ between the SpB and spine membranes of PCs. Closed and transparent circles indicate the mean values in each animal and cell, respectively, with colors indicating different animals. Black horizontal bars and error bars indicate the emmeans and 95% CIs, respectively. The NND_M-P_ was shorter in the spines than in the SpB membrane (real: 110.0 ± 5.6 nm, sim: 112.0 ± 5.6 nm, *n* = 22,391 values/140 compartments/7 mice, *p* < 0.001, Chi-LRT). n.s., not significant.

G-protein-coupled inwardly rectifying K^+^ (GIRK) channels are a family of lipid-gated potassium channels that are activated by PI(4,5)P_2_ and G-protein βγ-subunits (G_βγ_) released from G-protein coupled receptors (GPCRs; Whorton and MacKinnon, 2011). Thus, GIRK channels are expected to be associated with PI(4,5)P_2_ to be efficiently activated. To examine whether PI(4,5)P_2_ and GIRK channels are associated on dendritic membranes of mouse cerebellar PCs, we co-labeled PI(4,5)P_2_ and GIRK3, the most predominant subunit of GIRK channels in PCs (Aguado et al., 2008; Fernández-Alacid et al., 2009), on the P-face of the dendritic PC membranes. The gold particle labeling GIRK3 (GIRK3 particles) were observed throughout the dendritic SpBs and spines of the PCs (Fig. 10*A*). We compared the NNDs from GIRK3 to PI(4,5)P_2_ particles (NND_G-P_) between observed and simulated GIRK3 particles in spines and SpBs spines of PCs. The real NND_G-P_ in both spines and SpBs was significantly shorter than the simulated one (Chi-LRT: *p* < 0.001; Fig. 10*B*), suggesting that GIRK3 channels are associated with PI(4,5)P_2_ in the distal dendritic membranes of PCs. GIRK3 channels are also expressed on presynaptic PF boutons, including AZs (Fig. 10*A*; Fernández-Alacid et al., 2009; Luján et al., 2018b) in PF-PC synapses, and may regulate presynaptic excitability. In the presynaptic AZs, the real NND_G-P_ was significantly shorter than the simulated one (Chi-LRT: *p* < 0.001; Fig. 10*B*), indicating the association of GIRK3 with PI(4,5)P_2_. The mean NND_G-P_ in spine and AZ membranes was significantly shorter than that in SpB membranes (Fig. 10*C*), suggesting the tighter association of GIRK3 and PI(4,5)P_2_ in synaptic membranes.

#### The association of mGluR1α with PI(4,5)P_2_

Group I metabotropic glutamate receptors (mGluRs) are GPCRs coupled with Gα_q_ subunit and hydrolyze PI(4,5)P_2_ into inositol 1,4,5-trisphosphate (IP_3_) and diacylglycerol (DAG) through the activation of PLCβ. A subtype of Group I mGluRs mGluR1α is highly expressed on the dendritic shafts and spines but avoids PSDs in PCs, and forms clusters (L.J. Martin et al., 1992; Mateos et al., 2000; Mansouri et al., 2015; Luján et al., 2018a). To effectively produce the second messengers, PI(4,5)P_2_ clusters are expected to be located near the mGluR1α cluster on the dendritic membrane of PCs. To address this possibility, we co-labeled mGluR1α with PI(4,5)P_2_ on the dendritic membranes of PCs and compared the NNDs between mGluR1α and PI(4,5)P_2_ particles (NND_M-P_) with the fitted simulation of the mGluR1α particle distribution. The mGluR1α particles were distributed as clusters on the membranes of spines and SpBs (Fig. 10*D*,*E*) as previously reported (Luján et al., 2018a). The real NND_M-P_ was significantly shorter than the simulated one on the spine membrane (Chi-LRT: *p* < 0.001) but not on the SpB membrane (Chi-LRT: *p* = 0.36; Fig. 10*F*). These results suggest compartment-specific association of mGluR1α with PI(4,5)P_2_ on PC spines.

## Discussion

To understand the physiological roles of PI(4,5)P_2_ in neurons, it is essential to know its nanoscale distribution in the neuronal cell membranes. In this study, we examined the global and local density of PI(4,5)P_2_ using SDS-FRL and revealed that the PI(4,5)P_2_ density was different between subcellular compartments, rather than between cell types in the cerebellar cortex; in PCs, both of the global and local PI(4,5)P_2_ densities were higher at the distal dendrites than at the somatic and proximal dendrites; in GCs, the density was similar at the somata, dendrites, axons, and presynaptic boutons; in MLIs, the density of PI(4,5)P_2_ in clusters was lower in presynaptic bouton membranes than somatic and dendritic membranes. These heterogeneous PI(4,5)P_2_ distribution patterns may reflect different roles of PI(4,5)P_2_ in cell functions between different cell types.

### Visualization of PI(4,5)P_2_ distribution using SDS-FRL

To visualize phospholipids on cell membranes, aldehyde fixation is not suitable because they diffuse laterally even after fixation (K.A.K. Tanaka et al., 2010). The fluorescent protein-tagged PLCδ1-PH has been developed as a specific PI(4,5)P_2_ probe (Idevall-Hagren and De Camilli, 2015) and enabled single-molecule imaging on the plasma membrane of cultured cells using super-resolution microscopy (Wang and Richards, 2012; Milovanovic et al., 2016). However, in regions with densely packed proteins, e.g., AZs and PSDs, limited accessibility of the probes could hamper the PI(4,5)P_2_ visualization in brain tissues. Furthermore, the probe competes with endogenous PI(4,5)P_2_-binding proteins (Suh and Hille, 2008), making it difficult to quantify the PI(4,5)P_2_ dynamics. Our method solves these problems and is advantageous for visualizing the native PI(4,5)P_2_ distribution. First, phospholipids are physically immobilized by high-pressure freezing and carbon/platinum replication. In addition, since cytosolic proteins are removed by SDS treatment, PI(4,5)P_2_ are exposed on the replica surface, making high accessibility of the probe. Although our method has a limited linearity in high density ranges of PI(4,5)P_2,_ the labeling efficiency was constant up to 180 particles/µm^2^ (1000 PI(4,5)P_2_/µm^2^), covering most of our measurement ranges on neuronal membrane except those within the PI(4,5)P_2_ clusters.

### PI(4,5)P_2_ makes clusters on neuronal cell membranes in mouse cerebellum

How PI(4,5)P_2_ is distributed across cell membrane (clustered, randomly, or homogeneously) has been controversial. Previous studies demonstrated that PI(4,5)P_2_ forms clusters of 50–100 nm in diameter on the cell membrane of PC12 cells (Aoyagi et al., 2005; van den Bogaart et al., 2011; Wang and Richards, 2012) and mouse myoblast cells (Petersen et al., 2016). In contrast, a homogeneous distribution of PI(4,5)P_2_ on HEK293 cell membranes has been reported (van Rheenen et al., 2005). We found clustering of PI(4,5)P_2_-labeing particles on the HEK293 cell membrane, and this difference is probably because of the accurate 2D analysis on the replicas, indicating the superiority of our method.

The PI(4,5)P_2_ cluster area on the neuronal cell membrane was around 1000 nm^2^ (corresponding to 35 nm in diameter) in the cerebellar tissues. The mean area of the cluster was almost the same throughout the somatodendritic and axonal membranes and the neuronal cell types. In contrast, the cluster density and the intracluster particle density in the spine membrane are higher than in other somatodendritic compartments of PCs, supporting the contribution of PI(4,5)P_2_ to spine formation and morphologic long-term plasticity (Ueda and Hayashi, 2013; Lei et al., 2017). Clustering of PI(4,5)P_2_ indicates the importance of a spatial relationship between the clusters and effector proteins for their effective interaction, as discussed in the following section.

### PI(4,5)P_2_ distribution in the synaptic membrane

We demonstrated the accumulation of PI(4,5)P_2_ in the AZ of PF boutons, suggesting roles of PI(4,5)P_2_ on presynaptic activities. Rab3-interacting proteins (RIMs), tethering an SV to the AZ, syntaxin-1A, a member of the SNARE complex, and synaptotagmin-1, a calcium sensor on SVs, have affinity for PI(4,5)P_2_ (Aoyagi et al., 2005; Honigmann et al., 2013; Milovanovic et al., 2016; de Jong et al., 2018). Since Ca_V_2.1 directly binds to RIMs, the association of Ca_V_2.1 with PI(4,5)P_2_ supports the idea that PI(4,5)P_2_ anchors Ca^2+^ channels, SVs, and exocytic proteins for fast neurotransmitter release. PI(4,5)P_2_ also interacts with endocytic proteins, e.g., AP-2, AP180, and dynamin (T.F.J. Martin, 2001; Haucke, 2005; Posor et al., 2015). At calyx of Held synapses in rat brainstem, upregulation of PI(4,5)P_2_ by the retrograde nitric oxide signals accelerates vesicle endocytosis, which strengthens the homeostatic plasticity to maintain high-frequency synaptic transmission (Eguchi et al., 2012; Taoufiq et al., 2013). PI(4,5)P_2_ clusters may anchor the proteins involved in this pathway and work as their interaction site.

### The association of PI(4,5)P_2_ and ion channels and receptors

Since PI(4,5)P_2_ regulates the electrophysiological properties of several ion channels (Suh and Hille, 2008), clarifying spatial relationship between the ion channels and PI(4,5)P_2_ may provide insight into the regulation mechanism of neuronal excitability. In this study, we found ubiquitous association of Cav2.1 with PI(4,5)P_2_, not only in the AZ as described above, but also somatodendritic compartments of PCs, GCs and MLIs, indicating that the regulation of Cav2.1 function by PI(4,5)P_2_ could be extensive. GIRK3 was also ubiquitously associated with PI(4,5)P_2_ in dendritic membranes of PCs and AZs of PF boutons. Because PI(4,5)P_2_ is essential to activate GIRK channels by βγ subunit of G-protein (Whorton and MacKinnon, 2011), the association between GIRK3 and PI(4,5)P_2_ suggests that GIRK3 is constantly ready to be activated by G-protein coupled receptors. Since GIRK channels are also associated with GABA_B_ receptors at different compartments of the cerebellar neurons, such as dendritic shafts and spines of PCs and AZs of PF boutons (Fernández-Alacid et al., 2009; Luján et al., 2018b), the co-assembly of PI(4,5)P_2_, GIRK channels and GABA_B_ receptors may contribute to effective inhibitory postsynaptic transmission on PCs (Luján et al., 2018b).

In the dendritic shaft and spines of cerebellar PCs, mGluR1α couples with Gα_q_ subunit, PLCβ3 or β4, and IP_3_ receptors (IP3Rs) on the smooth endoplasmic reticulum (J. Tanaka et al., 2000; Nakamura et al., 2004; Nomura et al., 2007) to efficiently generate IP_3_ and DAG from PI(4,5)P_2_ following glutamate stimulation. However, the spatial correlation of PI(4,5)P_2_ and mGluR1α was still unclear. We found a significant association of mGluR1α with PI(4,5)P_2_ on the spine membrane in PCs, which may contribute to the effective production of the IP_3_/DAG in response to the mGluR1α activation. In contrast, no significant association of mGluR1α with the PI(4,5)P_2_ was observed in SpBs, though the application of mGluR1 agonist on the dendritic shaft induces calcium release from Ca^2+^ store (Hildebrand et al., 2009). Thus, the tight spatial association of PI(4,5)P_2_ as observed for Ca_V_2.1 and GIRK3 may not be critical for the coupling of mGluR1α activation and intracellular signal cascade depending on the compartments.

The kinetics of IP_3_ production by mGluR1, Gα_q_, and PLCβ have been investigated using caged-IP_3_ with calcium imaging and mathematical modeling (Finch and Augustine, 1998; Doi et al., 2005; Brown et al., 2008). Finch and Augustine estimated the intraspine IP_3_ concentration produced by 16 PF stimuli to be 10–20 μm based on the Ca increase induced by cage-IP_3_ uncaging (Finch and Augustine, 1998), indicating that 600–1200 IP_3_ is produced in the cytosol of a PC spine (0.1 µm^3^, Harris and Stevens, 1988). Mathematical modeling studies have shown that 70 μm (4200 molecules/spine) of IP_3_ is generated by repetitive PF stimulation (Hernjak et al., 2005; Brown et al., 2008). However, Brown et al., reported that the PI(4,5)P_2_ of 4000 molecules/µm^2^ in the spine membrane, the same as in neuroblastoma cells (Xu et al., 2003), could only produce up to 10 μm IP_3_, suggesting the requirement of a transient increase in PI(4,5)P_2_ to 10,000 molecules/µm^2^ through stimulus-dependent synthesis (Brown et al., 2008). In our study, since the density of PI(4,5)P_2_ particles in the spine is 70–100 particles/µm^2^, and assuming a labeling efficiency of 17% (Fig. 1*D*), the amount of PI(4,5)P_2_ in the spine (1.0 µm^2^, Harris and Stevens, 1988) can be estimated to be 400–600 molecules. This density is probably underestimated because the PI(4,5)P_2_ particles form clusters of ∼14,000 particles/µm^2^, causing marked reduction of the labeling efficiency. Thus, the amount of PI(4,5)P_2_ in the spine membrane may be sufficient for the IP_3_ production elicited by repetitive PF stimulation even in the resting state. Further studies with higher temporal resolution and mathematical modeling will be needed to analyze the precise dynamics of PI(4,5)P_2_ and IP_3_ on spine membranes induced by synaptic transmission.

In summary, we could successfully applied SDS-FRL to acute mouse cerebellar slices to analyze the nanoscale two-dimensional distribution of PI(4,5)P_2_ on the neuronal membrane, and its spatial relationship with GIRK3, Ca_V_2.1, and mGluR1α in PC dendrites and PF boutons. Notably, we have demonstrated a higher density of PI(4,5)P_2_ in distal PC dendrites, showing a specific association of mGluR1α with PI(4,5)P_2_ in spines. PI(4,5)P_2_ also showed concentration and association with Ca_V_2.1 and GIRK3 in presynaptic PF AZs. Although the spatial resolution and labeling efficiency at high PI(4,5)P_2_ densities need to be improved for more accurate quantitative distribution analysis, this method will help to elucidate the physiological role of PI(4,5)P_2_ in neuronal activity, including synaptic transmission and its long-term plasticity.
